# Targeting metabolic-epigenetic-immune axis in cancer: molecular mechanisms and therapeutic implications

**DOI:** 10.1038/s41392-025-02334-4

**Published:** 2026-01-26

**Authors:** Xing Wang, Xiyuan Luo, Ruiling Xiao, Xiaohong Liu, Feihan Zhou, Decheng Jiang, Jialu Bai, Ming Cui, Lei You, Yupei Zhao

**Affiliations:** 1 Department of General Surgery, Peking Union Medical College Hospital, Peking Union Medical College, Chinese Academy of Medical Sciences, Beijing, China; 2 Key Laboratory of Research in Pancreatic Tumor, Chinese Academy of Medical Sciences, Beijing, China; 3 National Science and Technology Key Infrastructure on Translational Medicine in Peking Union Medical College Hospital, Beijing, China

**Keywords:** Cancer metabolism, Cancer genetics, Tumour immunology, Tumour immunology

## Abstract

Cancer cells orchestrate a highly dynamic and interconnected network spanning metabolic, epigenetic, and immune mechanisms to drive adaptive plasticity and continuous development. This review synthesizes emerging insights into the coevolutionary strategies employed by malignant and stromal cells—particularly tumor cells and immune populations—across the continuum of tumorigenesis, metastasis, and treatment resistance. During tumor initiation, cancer cells rewire metabolism and generate oncometabolites that reshape the chromatin architecture to support immune evasion. Concurrently, metabolic competition in the tumor microenvironment (TME) induces epigenetic exhaustion of cytotoxic T cells, whereas tumor-associated myeloid cells adopt immunosuppressive and angiogenic phenotypes via metabolite-dependent histone modifications to promote carcinogenesis. At metastatic frontiers, under the local metabolic pressure of target organs, tumor cells undergo epigenetic reprogramming to evade immune attacks and support colonization. Premetastatic niches are preconditioned through exosome-mediated transfer of metabolic enzymes and noncoding RNAs that reprogram resident cells before tumor cells arrive. In cancer immunotherapy, tumors often exploit metabolic adaptative strategies to inhibit cell death signaling pathways or the compensatory activation of self-protective mechanisms to circumvent immune-mediated cytotoxicity and develop resistance to immunotherapy. By mapping these dynamic interactions, we propose a novel conceptual framework of the “metabolic-epigenetic-immune axis” that transcends traditional compartmentalized approaches and helps to identify nodal convergence points for therapeutic co-targeting. This review also prioritizes multitarget inhibitors arising from the convergence of metabolic reprogramming, epigenetic plasticity, and immune evasion networks. An integrated approach to these pathways advances next-generation precision oncology strategies aimed at circumventing the evolutionary resilience of cancer.

## Introduction

**Fig. 1:**
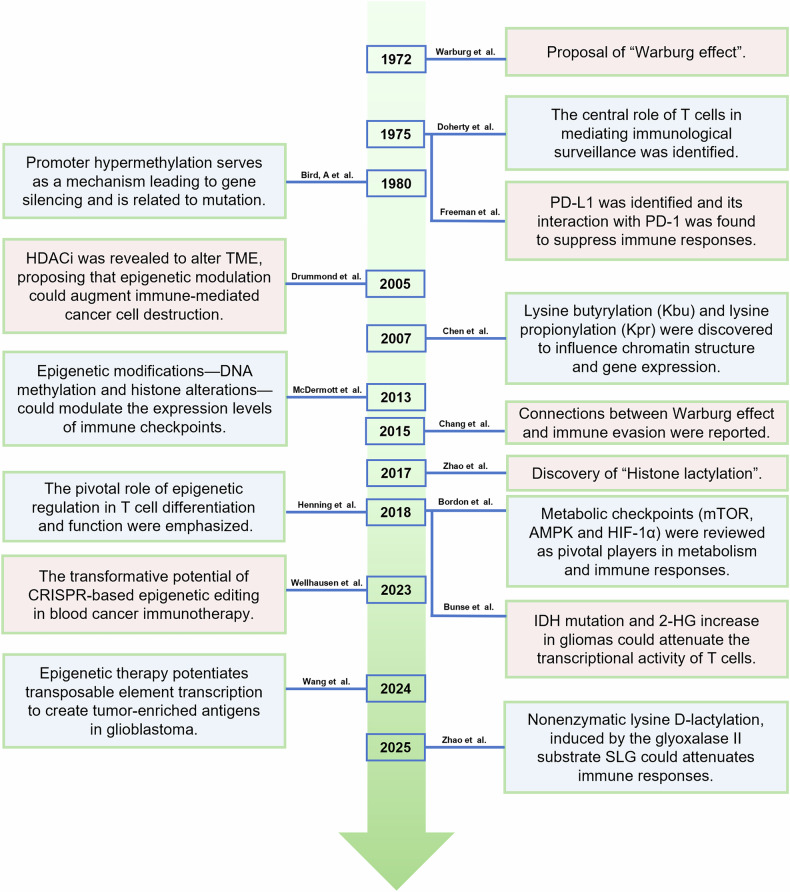
Retrospective summary of milestone events in the metabolic-epigenetic-immune regulation of cancer. The linear timeline shows outstanding contributions to the field in different eras. TME tumor microenvironment, HDACi HDAC inhibitor

Recent advances in cancer biology have highlighted the sophisticated strategies of cancer cells during progression. While much of the research to date has focused on individual mechanisms, including metabolic reprogramming,^1^ epigenetic alterations,^2^ and immune evasion,^3^ it is becoming clear that cancer cells use integrated, multipronged approaches to thrive in hostile environments, resist therapeutic interventions, and overcome host defenses.^4^ The chronological development of related research is illustrated in Fig. 1.

The advantage of this integrated strategy lies in the tumor’s capacity to orchestrate a cohesive, adaptable, and highly heterogeneous network. Even limited perturbations in metabolic pathways can induce profound epigenetic alterations, thereby amplifying immune evasion mechanisms.^5^ This amplification enables cancer cells to maximize their immune regulatory potential and minimize the metabolic burden within the TME. Moreover, the flexibility inherent in these multifaceted strategies equips cancer cells with the ability to respond dynamically to fluctuating immune pressures. As tumors progress, subclonal populations with distinct metabolic and epigenetic profiles emerge, which contributes to diversified survival tactics.^6^ These subclones can cooperate synergistically, leveraging their complementary capabilities to resist immune surveillance and facilitate survival.^7^ Consequently, it is increasingly difficult for the TME to be targeted with single-agent therapies. Immune-based therapies, such as immune checkpoint blockade (ICB), have shown promise in some cancers,^8^ but their effectiveness is often limited.^9^ An effective strategy to overcome these challenges may lie in combining immune therapies with metabolic and epigenetic interventions to reprogram the TME.

Collectively, these interconnected processes—the dynamic interplay of metabolic reprogramming, epigenetic modifications, and immune evasion—underscore the plasticity of cancer cells and present significant challenges in the development of effective therapeutic strategies. In this review, we explore the underlying regulatory mechanisms of this multifaceted approach and identify potential therapeutic targets to offer valuable insights for precision-based therapeutic frameworks.

## Historical overview and milestone events

### Early studies on metabolism, epigenetics, and immune regulation in cancer

#### Metabolism in cancer

Warburg’s seminal work in 1956 demonstrated that cancer cells preferentially utilize glycolysis over oxidative phosphorylation for energy production, a phenomenon now recognized as the Warburg effect, characterized by elevated lactate production.^10^ Further investigations by Mark et al. in the 1970s revealed diverse glycolytic phenotypes across cancer cell types, positioning glycolysis as a plastic survival mechanism.^11^ With advancements in cellular metabolism, studies in the early 21st century revealed that tumors exhibit distinctive metabolic profiles, including alterations in mitochondrial metabolism, such as the tricarboxylic acid (TCA) cycle and oxidative phosphorylation (OXPHOS),^12^ along with other key metabolic pathways, such as the pentose phosphate pathway,^13^ glutamine metabolism,^14^ lipid metabolism,^15^ amino acid metabolism,^16^ creatine metabolism,^17^ and pyruvate carboxylase-mediated processes.^18^ These metabolic adaptations not only support tumor cell growth but also reshape the TME, modulating immune cell function and facilitating immune evasion.

#### Epigenetics in cancer

Studies by Knudsen elucidated the critical role of CpG islands in genetic instability and cancer progression, highlighting how alterations in DNA methylation contribute to tumorigenesis.^19^ In the early 1980s, Bird identified promoter hypermethylation as a mechanism leading to gene silencing, marking a seminal discovery in the field of epigenetics.^20^ During the 1990s and 2000s, the understanding of epigenetic regulation advanced significantly. Modifications such as DNA methylation, histone modifications, and chromatin remodeling are pivotal in regulating gene expression in cancer.^21^ These findings underscore the idea that cancer-related genetic changes may arise not only from mutations but also from epigenetic alterations. These modifications drive oncogene activation and tumor suppressor gene silencing, thus playing a key role in cancer progression. Over the past decade, numerous chromatin-modulating enzymes have been identified, classified, and implicated in cancer.^22^ These enzymes are categorized into three main groups: “writers”, which add specific modifications to chromatin; “readers”, which recognize and interpret these modifications; and “erasers”, which remove specific modifications.^23^ Specifically, histone acetylation is conducted by histone acetyltransferase enzymes (HATs), and histone deacetylases (HDACs) define the removal of acetyl groups from an ε-N-acetyl-lysine amino acid on a histone.^24^ The balance between HDAC and HAT activities plays a crucial role in regulating histone acetylation levels and gene expression. Histones are methylated by enzymes, including arginine methyltransferases (PRMTs)^25^ and lysine methyltransferases (KMTs),^26^ and removed by lysine demethylases (KDMs).^27^ In short, these discoveries have continuously revolutionized our understanding of the role of epigenetic dysregulation driven by aberrant DNA methylation, histone modifications, and chromatin remodeling in cancer biology.

#### Immune regulation in cancer

In the 1970s, one of the earliest works proposed the concept of “immunological surveillance,” positing that the immune system plays a pivotal role in identifying and eliminating tumor cells.^28^ Expanding on this notion, Doherty and Zinkernagel’s work in 1975 emphasized the central role of T cells in mediating immunological surveillance.^29^ Freeman’s groundbreaking research later identified programmed death-ligand 1 (PD-L1), describing its interaction with the immune checkpoint receptor programmed cell death-1 (PD-1) and elucidating how PD-L1 negatively regulates lymphocyte activation by suppressing immune responses.^30^ In the 2000s, further advancements in tumor immunology illuminated several mechanisms through which tumors bypass immune surveillance, including immune checkpoint blockade, deficiencies in antigen presentation, and the secretion of immunosuppressive cytokines. Tumor cells actively modulate immune cell differentiation, function, and survival, thereby promoting immune tolerance.^31^ Collectively, these studies lay the groundwork for understanding how cancer cells manipulate immune responses, metabolism, and epigenetic pathways, revealing critical mechanisms underlying tumorigenesis and progression.

### Key research findings in the metabolic-epigenetic-immune regulation of cancer

#### Metabolite-mediated epigenetic modifications

Metabolites generated during cellular metabolism, including acetyl-CoA, S-adenosylmethionine (SAM), and alpha-ketoglutarate, serve as crucial cofactors for enzymes that regulate various epigenetic modifications. These metabolites influence DNA methylation, histone modifications, and chromatin remodeling, ultimately modulating gene expression, particularly in tumor cells. In recent years, novel types of histone modifications have gained considerable attention for their roles in gene regulation and disease processes.

Metabolites derived from glucose metabolism, including key intermediates such as acetyl-CoA, succinyl-CoA, lactyl-CoA, and other compounds from glycolysis and the citric acid cycle, function as critical substrates for various acylation modifications. Succinylation, first proposed in 2011, involves the addition of a succinyl group (C₄H₆O₄) to lysine residues on histones, thereby modulating chromatin structure and gene expression.^32^ Subsequent investigations, particularly post-2015, illuminated the connection between succinylation and cellular energy metabolism, suggesting its involvement in the pathophysiology of cancer and metabolic disorders.^33^ Lactylation, a novel histone modification identified in 2017, occurs when a lactyl group (C₃H₆O₃) is enzymatically appended to histone lysines.^34^ This modification not only regulates gene transcription but is also intricately linked to cellular energy metabolism, particularly in the contexts of inflammation and cancer.^35^ In 2021, significant advancements in the understanding of lactylation were made, culminating in its recognition as a critical regulator of metabolic reprogramming in immune cells.^36^ By 2024, research has firmly established lactylation’s pivotal role in both cellular metabolism and immune response modulation.^37–39^ Recent studies in 2025 have shown that nonenzymatic lysine D-lactylation, induced by the glyoxalase II substrate SLG, attenuates immune responses.^40^ These canonical metabolites exert their downstream effects by serving as critical substrates for epigenetic modifications, thereby bridging cellular metabolic states with chromatin dynamics.

Moreover, intermediates from fatty acid synthesis and degradation also contribute to acylation modifications. Palmitoylation, a modification involving the covalent attachment of palmitoyl-CoA to histone lysines, was characterized in 2007.^41^ Further studies underscored its notable implications for cellular metabolism and tumor metastasis.^42^ Lysine butyrylation (Kbu), identified along with propionylation (Kpr) in 2007, refers to the attachment of butyryl-CoA to histones, influencing chromatin structure and gene expression.^43^ By 2020, accumulating evidence highlighted the role of butyrylation in immune tolerance, anti-inflammatory responses, and cancer immunotherapy.^44^ Histone malonylation, first proposed in 2012, suggests that malonyl-CoA, an intermediate in fatty acid metabolism, not only plays a role in fatty acid synthesis but also participates in histone modification through specific enzymatic catalysis.^45^ Studies in 2015 highlighted the role of malonylation in tumor cell metabolism, particularly in terms of mitochondrial function and FAO.^46^ Lysine 2-hydroxyisobutyrylation (Khib) was discovered through MS/MS and HPLC coelution experiments in 2014,^47^ and Khib-modulated glycolytic enzymes are essential for regulating glycolysis in response to nutrient availability.^48^ β-hydroxybutyrate, a ketone body produced during fasting or exercise, participates in β-hydroxybutyrylation, a modification in which β-hydroxybutyrate binds covalently to histone lysines. First identified in 2016,^49^ this modification has been associated with reprogramming of tumor metabolism, particularly glycolysis.^50^ In addition, intermediates from lysine and tryptophan contribute to acylation modifications. Lysine glutarylation (Kglu) was first detected and comprehensively validated in 2014,^51^ to modulate the regulation of gene expression and metabolism and may be highly correlated with multiple biological functions.^52^ Crotonylation, discovered in 2011, involves the addition of crotonyl-CoA to histone lysines, thereby impacting gene expression and cell cycle regulation during tumor development.^53^ These modifications dynamically link nutrient availability and metabolic flux to chromatin remodeling and transcriptional regulation, enabling cells to adapt to environmental cues.

In addition to well-established acylation modifications, emerging modifications such as oxidation,^54^ cinnamoylation and oleoylation^55^ have garnered increasing attention owing to their associations with cellular metabolism, epigenetic regulation, and disease progression. These findings collectively underscore the pivotal role of metabolic pathways in modulating epigenetic modifications, highlighting their potential as therapeutic targets in the treatment of cancer and other metabolic disorders.

#### Epigenetic modulators and immune response

Epigenetic research has progressively underscored its critical role in cancer immunology and therapy, with several key developments shaping its trajectory. In 2005, Drummond and Noble identified the potential of histone deacetylase inhibitors (HDACis) to alter the tumor microenvironment, suggesting that epigenetic modulation could augment immune-mediated cancer cell destruction.^56^ This discovery catalyzed the development of epigenetic-based therapies aimed at reprogramming immune responses to cancer. A significant breakthrough occurred in 2013, when McDermott and Atkins examined the role of the immune checkpoint PD-1 in cancer, suggesting that epigenetic modifications—such as DNA methylation and histone alterations—could modulate the expression of immune checkpoints, thereby providing insights into tumor-mediated immune evasion.^57^ Henning and Roychoudhuri (2018) further revealed that the epigenetic control of CD8 + T cell differentiation could enhance T cell functional plasticity, offering a mechanistic rationale for improving immunotherapy efficacy.^58^ In 2021, the transformative potential of CRISPR-based epigenetic editing in immune cells was explored, revealing that targeted epigenetic modifications could optimize immune cell function, suggesting an innovative approach for cancer treatment.^59^ Further investigations revealed that epigenetic reprogramming in various immune cells, such as polymorphonuclear myeloid-derived suppressor cells (MDSCs), the major infiltrating immune cell type that causes immune evasion in prostate cancer, contributes to an immunosuppressive microenvironment.^60^ The integration of epigenetic therapies with immunotherapies, including ICIs and CAR-T cell therapies, has gained significant momentum in clinical research. Yu and Zhao (2024) reviewed the application of these combined strategies in clinical practice,^61^ emphasizing the increasing incorporation of epigenetic approaches into cancer treatment regimens and underscoring the promising future of epigenetic immunotherapy.

#### Metabolic checkpoints and immune regulation

Early investigations into the TME highlighted how metabolic alterations influence immune cell functions. A seminal contribution in 2015 by Chang et al. established a connection between the Warburg effect and immune cell activity. Their work demonstrated that tumor cells manipulate immune responses by altering the metabolic landscape of T cells and macrophages.^62^ This insight spurred further research into the role of oncometabolites, particularly 2-hydroxyglutarate (2-HG), in epigenetic regulation. In 2018, Bunse et al. revealed that isocitrate dehydrogenase (IDH) mutation and 2-HG accumulation in gliomas perturbed the transcriptional activity and polyamine biosynthesis of nuclear factors in activated T cells, resulting in the suppression of T cell activity.^63^ In addition to evidence of oncometabolites, increasing evidence underscores the intersection between metabolic reprogramming and immune checkpoint regulation. Researchers have identified how metabolic disturbances—such as glucose deprivation, lactate accumulation,^64^ and hypoxia.^65^—within the TME modulate immune checkpoint expression. Concurrently, metabolic checkpoints such as mammalian target of rapamycin (mTOR), AMPK, and hypoxia-inducible factor (HIF)-1α are recognized as pivotal players in both cellular metabolism and immune responses. Specifically, a landmark study by Pearce et al. elucidated the role of metabolic pathways in T cell differentiation and function, emphasizing the central role of mTORC1 activation in T cell metabolism during immune responses.^66^ Conversely, the activation of AMPK, which acts as a metabolic sensor, was shown to enhance antitumor immunity by inhibiting mTOR signaling and promoting autophagy, thereby strengthening immune surveillance.^67^ By 2020, the focus shifted toward therapeutic strategies targeting metabolic pathways to improve the efficacy of cancer immunotherapy. Studies have demonstrated that metabolic reprogramming of T cells could increase the effectiveness of ICIs, prompting widespread interest in combining metabolic modulators with immunotherapy to overcome tumor immune resistance.^68^ By 2021, clinical investigations into the use of metabolic reprogramming agents alongside ICIs and other immunotherapies yielded promising results.^69^ These studies demonstrated enhanced antitumor immunity and improved patient outcomes, emphasizing the potential of metabolic interventions to advance cancer immunotherapy.

#### Epigenetic reprogramming-mediated metabolic remodeling

Epigenetic alterations actively participate in the metabolic reprogramming of cancer. Increased histone and DNA methylation marks transcriptionally repress fructose-1,6-biphosphatase (FBP1), which triggers the reprogramming of glucose metabolism to sustain cancer stem cell-like properties in breast cancer cells.^70^ In parallel, accumulating evidence suggests that protein acetylation plays a major regulatory role in many facets of the transcriptional control of metabolism.^71^ Furthermore, the roles of noncoding RNAs (ncRNAs) in metabolic reprogramming have been shown to encompass both transcriptional and posttranscriptional regulation.^72^ These findings emphasize the intricate nature of epigenetic regulation in metabolism, suggesting that a deeper exploration of ncRNA-mediated epigenetic mechanisms could dramatically expand the list of potential drug targets. As our understanding of the role of epigenetic reprogramming in metabolic changes continues to evolve, it has catalyzed the development of novel cancer therapies that target both metabolic pathways and epigenetic regulators.^73^

### Biological foundations of metabolic, epigenetic and immune integration

The interplay between metabolic alterations and epigenetic modifications in cancer plays a pivotal role in initiating immune cascades that can either promote or impede tumor progression. Metabolites and enzymes produced through disrupted cellular metabolism can directly influence epigenetic regulation within the nucleus, thereby modulating immune cell recruitment, activation, and differentiation. Metabolic processes and epigenetic modifications commonly occur within distinct cellular compartments, ensuring the orderly execution of various biological functions. We discuss the current understanding of the molecular mechanisms underlying metabolic enzyme-mediated and metabolite-mediated modulation of chromatin. These processes promote metabolic and epigenetic integration and necessitate the active transport of metabolites and metabolic enzymes into the nucleus.

#### Metabolite-mediated modulation of gene expression

One of the most extensively studied chromatin modifications involves the addition of a methyl (-CH3) group to the ε-amino group of lysine or arginine residues on histones, as well as to CpG islands in DNA.^74^ This modification is derived from the metabolism of the essential amino acid methionine (Met), which, in mammals, is acquired primarily through dietary sources.^75^ Upon uptake, Met is converted into the methyl-donor metabolite S-adenosylmethionine (SAM), which serves as a substrate for DNA and histone methyltransferases.^76^ The key histone methylation marks linked to this metabolic pathway include H3K4me3,^77^ H3K9me1/2/3,^78^ H3K27me3,^79^ and H3K36me3,^80^ which are associated with differential gene expression patterns. Metabolites such as NAD+ and pyruvate can undergo mitochondrial-to-nuclear shuttling, where they are transported from the mitochondria to the nucleus, thereby influencing nuclear processes in various ways, including regulating various enzymes as important cofactors, providing substrates for acylation or directly triggering receptors or kinases to initiate signal cascades.^81^ Acetyl-CoA, the product of pyruvate, has long been recognized as an active substrate for histone acetylation,^82^ and elevated NAD+ levels can increase the activity of sirtuins, which are NAD + -dependent deacetylases that promote histone deacetylation.^83^ In addition to histone acetylation, other protein acylation modifications are described in detail in the key research findings section according to their order of discovery.

Moreover, cells have the capacity to detect metabolic changes and initiate a cascade of signaling events referred to as metabolite sensing, which regulate cell signaling pathways and gene expression.^84^ This process serves as a vital link between the extracellular environment and cellular function, enabling cells to rapidly perceive environmental fluctuations and reorganize their metabolic networks accordingly.^85^ GPR31, a G protein-coupled receptor (GPCR), recognizes intermediates of the TCA cycle.^86^ Recent studies have demonstrated that lactic acid and pyruvate can act as potent inducers of GPR31-mediated dendritic processes in intestinal phagocytes, potentially enhancing immune responses.^87^ In addition, succinate receptor 1 (SUCNR1), also known as GRP91, is activated by succinate, which plays a pivotal role in modulating macrophage behavior within the TME.^88^ Additionally, α-KG increases the mRNA level of the transcription factor *Tbet*,^89^ and enhances mTORC1 signaling.^90^ to direct naive T cells toward Th1 cell differentiation.

**Fig. 2:**
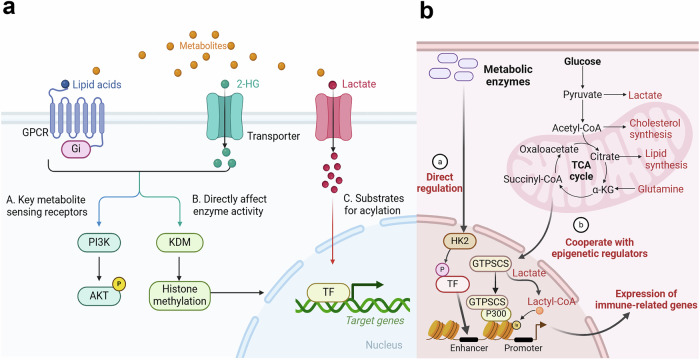
Biological foundations of metabolic and epigenetic integration. The interplay between metabolism and epigenetics can be classified into two primary mechanisms: metabolite-driven (a) and enzyme-driven (b) activation. **a**. Metabolites can directly suppress the activity of epigenetic regulators, act as substrates for acetylations, or trigger signaling cascades through metabolic sensing pathways to initiate gene expression. **b**. Metabolic enzymes can perform noncanonical functions to activate transcription or form complexes with classical “writers”, collectively driving the formation of acetylations. This dual mechanism underscores the intricate relationship between metabolic processes and epigenetic regulation in immune-related gene expression control. The figure was generated with BioRender (https://biorender.com). TF transcription factor, TCA tricarboxylic acid cycle

Notably, GPCRs also play pivotal roles in metabolite-sensing mechanisms related to fatty acids, serving as the primary receptors for these metabolites.^91^ Specifically, GPR43 preferentially recognizes short-chain fatty acids such as acetate and propionic acid. Activation of GPR43 impairs the function of CD8 + T cells and leads to excessive activation of dendritic cells (DCs), ultimately promoting colorectal cancer (CRC) development.^92^ GPR84, a receptor for medium-chain fatty acids (C9–C14), including capric acid (C10), undecanoic acid (C11), and lauric acid (C12),^93^ has been shown to increase macrophage phagocytosis of APMAP-deficient cancer cells.^94^ Additionally, mTOR is a key regulator of amino acid sensing and is implicated in various cancers.^95^ Kynurenine can be sensed by both aryl hydrocarbon receptor (AhR) and GPR35, which suppress immunosurveillance and play a regulatory role in colonic tumorigenesis.^96^ These studies reveal how metabolite-sensing mechanisms—via GPCRs or AhR/GPR35—integrate metabolic cues with immune and oncogenic signaling. Targeting these metabolite‒receptor axes could disrupt tumor‒immune crosstalk, offering combinatorial therapeutic strategies against cancer (Fig. 2a).

#### Metabolic enzyme-mediated epigenetic reprogramming

Metabolic processes and epigenetic modifications are intricately linked, with metabolic enzymes playing multiple roles in influencing gene expression. Some enzymes can translocate to the nucleus under metabolic stress and cooperate with epigenetic regulators (writers or erasers) to modulate gene expression (Fig. 2b). LDHA (lactate dehydrogenase A), for example, catalyzes the conversion of pyruvate to lactate, which serves as a major epigenetic carbon source for histone acetylation.^97^ Moreover, nuclear GCDH (glutaryl-CoA dehydrogenase) in conjunction with CBP (CREB-binding protein) plays a critical role in regulating lysine crotonylation (Kcr),^5^ and ALDOB/KAT2A interactions and KAT2A coupled with the α-ketoglutarate dehydrogenase (α-KGDH) complex function to form H3K9ac,^98^ and histone H3 succinylation, respectively.^99^ Similarly, ACSS2 and KAT2A,^39^ CBX3-P300^100^ and GTPSCS coupled with P300 function in lysine lactylation.^101^

The sirtuin family of proteins, particularly SIRT1, is another example of how metabolism and epigenetics are coupled. Sirtuins regulate various metabolic pathways^102^ while also influencing epigenetic processes such as histone deacetylation.^103^ In addition, metabolic enzymes that localize to the nucleus, including pyruvate kinase M2 isoform (PKM2), 6-phosphofructo-2-kinase/fructose-2,6-bisphosphatase 4 (PFKFB4), FBP1 and glyceraldehyde-3-phosphate dehydrogenase (GAPDH), α-KGDH and fumarase, inosine 5′-monophosphate (IMP) dehydrogenase (IMPDH) and GMP synthase (GMPS), and MAT2A are independent of the production of metabolites to directly mediate histone modifications and promote transcription regulation.^101^ These interactions exemplify how metabolic shifts can drive epigenetic changes that balance cellular energy production with efficient gene expression to maintain optimal cellular functions.

#### Metabolic triggers of immune dynamics

Within the complex TME, metabolic regulation of immunity operates through two distinct yet interconnected axes. The first axis, termed immunometabolism, encompasses cell-intrinsic metabolic reprogramming within immune cells that directly governs their differentiation, activation and functional programming. Immunometabolism is dynamically regulated through the interplay of serine/threonine kinases, immunological cues and nutrient signaling networks, including phosphoinositide 3 kinase (PI3K) — protein kinase A, G and C (AGC) kinases, mechanistic target of rapamycin (mTOR) and liver kinase B1–5′ AMP-activated protein kinase (LKB1–AMPK) signaling, especially in T cells.^104^ Phosphoinositide 3-kinase (PI3K) functions in converting phosphatidylinositol-(4,5)-bisphosphate (PIP2) to phosphatidylinositol-(3,4,5)-trisphosphate (PIP3), a crucial step for the recruitment of proteins with pleckstrin homology (PH) domains, such as phosphoinositide-dependent kinase 1 (PDK1) and Akt.^105^ PDK1 can sustain glucose uptake and glycolysis in response to interleukin-2 (IL-2) stimulation.^106^ Akt, a key AGC kinase extensively studied in immune cells, facilitates glycolysis during the activation process of the T-cell receptor (TCR) and costimulatory receptor.^107,108^ Furthermore, glycogen synthase kinase 3 (GSK-3), a serine/threonine kinase involved in Akt signaling, affects the survival and activation of T and B cells. Active GSK-3 restricts nuclear factor of activated T cells (NFAT) activity, thus modulating immune cell function.^109^ Moreover, mTOR serves as a key metabolic sensor, with mTORC1 orchestrating transcriptional programs and TF expression levels, including c-Myc, which increases aerobic glycolysis, glutaminolysis, and mitochondrial metabolic remodeling to regulate the proliferation and differentiation of Teff cells.^90,108^ Additionally, mTORC1-dependent activation of mitochondrial metabolism supports key metabolites—such as α-ketoglutarate, 2-hydroxyglutarate, and acetyl-CoA—necessary for T cell epigenetic programming.^110,111^ A further outcome of enhanced mitochondrial biogenesis is the increased activity of serine- and folate-dependent one-carbon metabolism, which is crucial for T cell activation.^112,113^ Furthermore, the LKB1-AMPK signaling pathway enables metabolic flexibility in T cells under conditions of energy stress, thereby promoting the activation and response of Teff cells. AMPK enhances T cell survival by inhibiting lipid biosynthesis pathways,^114^ while simultaneously supporting glutaminolysis and OXPHOS,^115^ to sustain intracellular ATP levels. In addition, AMPK regulates mitochondrial homeostasis through PGC-1α-mediated mitochondrial biogenesis,^116^ a process that contributes to the antitumor functionality of T cells.^117^ Immunometabolic signaling networks are intricately interconnected and reciprocal, collectively driving the metabolic regulation required to fulfill context-specific demands for cellular function. In addition, how key immunometabolic checkpoints, such as IDO1, ACAT, and MTHFD2, affect the function and fate of immune cells will be discussed in the next section.

The second axis involves metabolic adaptations within tumor cells, which not only fuel malignant proliferation but also systemically dysregulate antitumor immunity. Accumulating evidence reveals that cancer cells exploit metabolic rewiring to evade immune surveillance through multiple mechanisms, including the suppression of immunogenicity, metabolic competition for nutrients (e.g., glucose and glutamine), checkpoint ligand modulation, and the secretion of oncometabolites that impair immune cell function. These tumor-imposed metabolic constraints create a self-reinforcing immunosuppressive niche, and we dissect these dual regulatory layers in the following sections.

#### Nuclear import mechanism

The entry of metabolites or metabolic enzymes into the nucleus is a prerequisite for the initiation of metabolic-epigenetic-immune regulation. Metabolites and other water-soluble small molecules can enter the cell nucleus through direct diffusion. Nuclear localization signals (NLSs), short peptide sequences enriched in arginine and lysine residues, serve as critical recognition motifs for nuclear import.^118^ Substantial experimental evidence suggests that NLS‒receptor interactions orchestrate the precise docking and trafficking of nucleus-targeted proteins through the nuclear transport machinery.^119^ Additionally, active transporters utilize ATP to drive the movement of molecules across membranes,^120^ and SNARE proteins also facilitate the transport of specific molecules by recognizing their target substrates, ensuring precise molecular localization and function.^121^ This coordinated trafficking not only establishes the molecular foundation for initiating chromatin remodeling and transcriptional regulation but also highlights the nuclear transport machinery as potential therapeutic targets to disrupt pathogenic metabolic signaling in cancer.

## Integrated signaling networks involved in metabolic-epigenetic-immune regulation

### First contact: a self-nuclear response to environmental changes

**Fig. 3:**
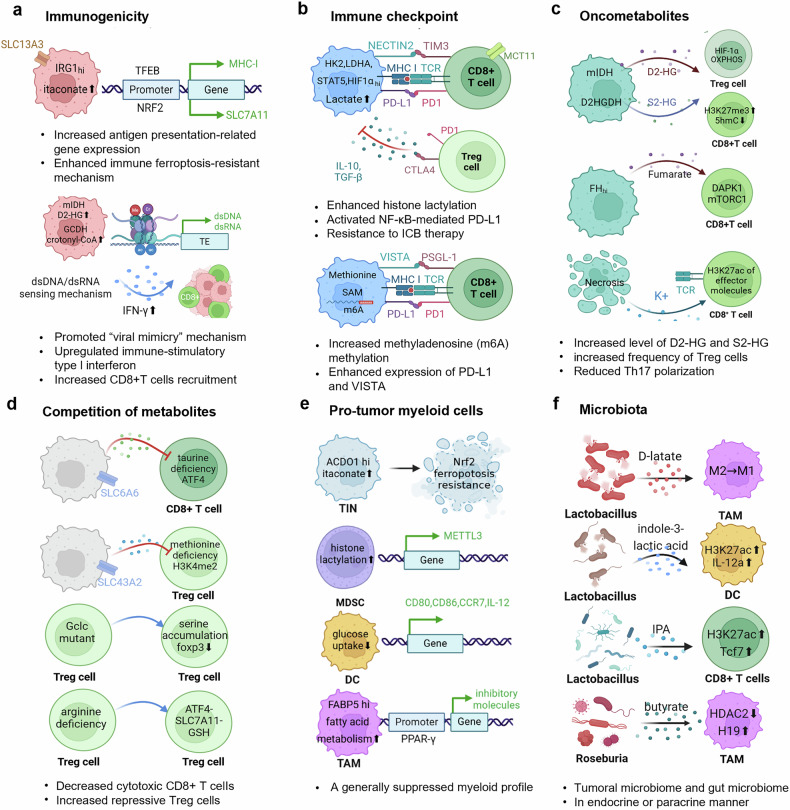
Metabolic-epigenetic-immune strategies within the TME. The first category focuses on cancer cell-intrinsic alterations, including the modulation of immunogenicity (**a**) and the expression of immune checkpoint molecules (**b**). The second category, centered on immune cells, encompasses strategies such as metabolic competition (**d**), the diffusion of oncometabolites (**c**), and phenotypic plasticity (**e**). Moreover, the increasing recognition of the microbiome’s influence on the tumor microenvironment is also important (**f**). The figure was generated with BioRender (https://biorender.com). TME tumor microenvironment, Treg regulatory T cell, TAM tumor-associated macrophage, DC dendritic cell, MDSC myeloid-derived suppressor cell, TIN tumor-infiltrating neutrophil, TAM tumor-associated macrophage, TE transcriptional element, ICB immune checkpoint blocker

Tumors employ a range of strategies to evade immune surveillance, including the upregulation of inhibitory checkpoints (Fig. 3b), the production of oncometabolites (Fig. 3c) and the competition of metabolites (Fig. 3d), which suppress T cell cytotoxicity. In addition, they downregulate antigen presentation pathways, impairing the ability of DCs to present tumor antigens to reduce immunogenicity (Fig. 3a) and recruiting and training immune cells (Fig. 3e) to form a suppressive TME. To achieve these goals, metabolic reprogramming serves as an accessible and critical approach.

#### Oncogene-driven mutagenesis and antigen release

Oncogene-driven nutrient uptake, particularly through *MYC* and *RAS*, leads to the accumulation of mitochondrial reactive oxygen species (ROS), which, in conjunction with chromatin remodeling, elevates mutagenesis rates.^122^ This process also triggers cellular senescence and the secretion of inflammatory factors, which subsequently activate immune responses.^123^ Conversely, tumors can evade immune surveillance by stabilizing tumor-intrinsic antioxidant pathways, such as through mutations that inactivate Kelch-like ECH-associated protein 1 (KEAP1) or stabilize nuclear factor erythroid 2-related factor 2 (NRF2), rendering them resistant to ICB.^124^ Furthermore, dysregulation of the urea cycle (UCD), often driven by *MYC* activation,^125^ reroutes nitrogen toward pyrimidine biosynthesis,^126^ thereby promoting the generation of hydrophobic tumor antigens and enhancing tumor immunogenicity. Collectively, these findings indicate that metabolic alterations, including oncogene-induced ROS production and UCD, not only facilitate tumor growth but also contribute to increased mutagenesis and enhanced immunogenicity.^127^

#### Alterations in immunomodulatory ligands

Cancer cells exhibit classical features of the Warburg effect, which plays a crucial role in immune evasion.^128^ The initial step in glycolysis is catalyzed by hexokinase (HK), which phosphorylates glucose to form glucose-6-phosphate (G-6-P). The overexpression of HK2 and enhanced aerobic glycolysis have been observed in various cancers,^129^ with HK2 functioning as both a glucose sensor and a protein kinase that regulates the nuclear factor-κB-gene binding (NF-κB)-mediated transcription of CD274 (encoding PD-L1), thereby promoting immune evasion.^130^ The final step of glycolysis is catalyzed by LDHA, which has been implicated in tumor progression and is involved in the formation of histone lactylation.^131^ The LDHA-histone lactylation-Nectin2 axis has been identified as a key positive feedback loop in the metastasis of PDAC.^132^ In addition to glycolytic enzymes, transcription factors such as STAT5 contribute to lactate production and immune suppression by promoting the nuclear translocation of E3 ubiquitin-binding protein (E3BP) and facilitating histone lactylation, which in turn enhances PD-L1 transcription.^133^ ACSS2, KAT2A,^39^ and CBX3-P300,^100^ which are unidentified lactyl-CoA synthetases and lactyltransferases, respectively, are involved in gene expression and immune evasion. Specifically, H3K18 lactylation supports immunosuppression by inducing POM121 to increase MYC activity in NSCLC^37^ and PD-L1 expression in gastric^134^ and prostate cancer.^135^ Therefore, histone lactylation serves as a crucial mechanism by which glycolysis influences immunomodulatory ligands (Fig. 3b).

Beyond glycolysis,^136^ tumors may rely on flexible fuel choices, such as OXPHOS, fatty acids,^137^ ketone bodies,^138^ cholesterol metabolism,^139^ amino acid oxidation,^140^ and nucleotide metabolism,^141^ allowing cancer cells to adopt various strategies to remodel the expression of PD-L1 for immune evasion.^142^ Nicotinamide phosphoribosyltransferase (NAMPT), the rate-limiting enzyme of NAD+ biogenesis, drives interferon γ (IFNγ)-induced PD-L1 expression in multiple types of tumors and governs tumor immune evasion in a CD8 + T cell-dependent manner.^141^ Kynurenine (Kyn) produced from tryptophan transcriptionally regulates the expression of Siglec-15 via AhR, and the overexpression of Siglec-15 promotes immune escape by suppressing T cell infiltration and activation.^143^ In addition, neoplastic cells exhibit major rewiring of lipid metabolism in support of immune evasion.^144^ Increased fatty acid catabolism in GBM cells caused by increased lipid flux through fatty acid oxidation (FAO) leads to immune evasion via the upregulation of CD47, referred to as the “don’t eat me” signal.^145^ In addition, the tumor-specific folate cycle enzyme methylenetetrahydrofolate dehydrogenase 2 (MTHFD2) drives the folate cycle to sustain sufficient uridine-related metabolites, including UDP-GlcNAc, which promotes PD-L1 transcription.^146^ S-Adenosylmethionine derived from methionine metabolism promotes N6-methyladenosine (m6A) methylation and the translation of immune checkpoint molecules, including PD-L1 and VISTA,^147^ further contributing to immune escape in cancer cells.

#### Suppression of antigen-presenting cell recruitment

The release of cancer-associated antigens (CAAs) as well as intracellular metabolites could increase antigen-presenting cell (APC) activation and antigen presentation to cancer antigen-specific T cells. Cancer cells have developed strategies to prevent antigen-presenting processes. Increased expression of cis-aconitate decarboxylase (IRG1) and the subsequent production of tumor-intrinsic itaconate have been shown to increase antigen presentation by promoting the nuclear translocation of transcription factor EB (TFEB).^148^ In contrast, itaconate produced by TAMs dampens tumor immunogenicity through the SLC13A3-itaconate-NRF2-SLC7A11 signaling axis.^149^ The accumulation of lipids in ovarian cancer cells driven by the upregulation of fatty acid synthase (FASN) has been shown to impair the cross-priming activity of tumor-infiltrating DCs.^150^ However, DAMPs such as ATP initially facilitate the recruitment of DCs, primarily via the activation of P2Y2 purinergic receptors, and trigger downstream immune responses.^151^ Tumors exploit mechanisms such as ATP catabolism by ectonucleotidases, such as CD39, CD73 and A2B receptor signaling, to induce a tolerogenic DC phenotype characterized by the expression of immunosuppressive factors such as arginase 2 (ARG2) and indoleamine 2,3-dioxygenase 1 (IDO1).^152^

In addition to affecting the activation and recruitment of APCs, cancer cells can also interfere with the major histocompatibility complex-I (MHC-I) antigen presentation pathway by downregulating MHC-I positive regulators or upregulating MHC-I negative regulators through various mechanisms^153,154^ (Fig. 3a). Current research posits that the downregulation of MHC-I presentation in most tumors is due primarily to mutations or deletions in key genes, including but not limited to Traf3, B2M, TAP1/2, and JAK.^155^ The Shadel group reported that treating wild-type cells with succinate was sufficient to inhibit lysine-specific demethylase (LSD) activity, thus increasing cell surface MHC-I and MHC-APP gene expression.^156^ Conversely, when glutamine, the primary source of succinate, was depleted, succinate accumulation in cells was significantly inhibited, thereby reducing MHC-I and MHC-APP gene expression. Furthermore, synthesized C_4_H_4_Na_2_O_4_ NPs can release high concentrations of Na^+^ and succinate ions into tumor cells, leading to an increase in intracellular osmolarity, which inhibits tumor immune escape through the upregulation of MHC-I expression.^157^ Moreover, in preclinical models of HCC, ablation of Arf1, which encodes a core regulator of fatty acid metabolism,^158^ preserves the mitochondrial checkpoint and inhibits ISR signaling, leading to increased tumor infiltration by DCs and restored anticancer immunity.^159^ Targeting metabolic nodes could restore MHC-I expression, enhance DC infiltration, and reverse immunosuppression, which highlights the therapeutic potential of metabolic interventions to rewire immune recognition and combat immune escape in cancers.

#### Impaired viral mimicry and suppressed antigenicity

Increasing evidence suggests that “viral mimicry” – a process in which tumor cells activate endogenous retroviral elements or transposable repeats, thereby mimicking viral infection through double-stranded RNA (dsRNA) production – plays a pivotal role in shaping tumor immunogenicity and initiating innate immune responses characterized by type I interferon upregulation and viral defense gene activation^133^ (Fig. 3a). This process involves the derepression of transposable elements (TEs)—vestigial viral sequences scattered throughout the genome—that are normally silenced but become activated under epigenetic reprogramming, triggering immune sensors such as MDA5, RIG-I, and MAVS (for dsRNA) and cGAS and STING (for dsDNA).^153^ Inhibitors targeting epigenetic regulators such as DNA methyltransferases (DNMTs), KDM5B,^160^ LSD1,^161^ and SETDB1^162^ can directly modulate DNA methylation, a major mechanism of epigenetic silencing to increase type I interferon signaling and improve responses to immunotherapy through the activation of immunogenic elements derived from TEs. In addition, the inhibition of mutant IDH1 (mIDH1) restores DNA demethylation, leading to the derepression of cGAS and specific TE promoters. This results in the production of dsDNA, which activates the cGAS-STING-IRF3 pathway, thereby triggering viral mimicry and promoting immune surveillance.^153^ In addition to DNA modifications, the loss of histone lysine crotonylation mediated by GCDH has been shown to increase the generation of cytosolic dsRNA and dsDNA, resulting in increased MDA5 and cGAS activation, thus increasing type I interferon signaling. This response compromises glioblastoma stem cell tumorigenicity and promotes CD8 + T-cell infiltration.^5^ Methionine restriction has also been shown to promote cGAS activation by interfering with its inactivating methylation by SUV39H1.^163^ Similar results have been reported in CRC cells deprived of both endogenous and exogenous sources of the nonessential amino acid serine, resulting in restored sensitivity to ICIs.^164^ Additionally, the inhibition of arginine methyltransferase leads to the retention of introns, forming dsRNA that activates immune receptors and upregulates the interferon pathway, triggering an endogenous immune response and inducing cell death.^165^ Collectively, metabolic regulation serves as a critical initiator of “viral mimicry” by reshaping the epigenetic landscape to derepress transposable elements, thereby generating endogenous nucleic acids that activate immune sensors.

#### Abnormal release of chemokines

Chemokines are essential for orchestrating the recruitment of immune cells, thereby shaping the formation and dynamics of the TME.^166^ The deficiency of quinoid dihydropteridine reductase (QDPR), an essential enzyme in biopterin metabolism, results in the accumulation of dihydrobiopterin (BH2) and a concomitant reduction in the tetrahydrobiopterin (BH4)/BH2 ratio in PDAC. This imbalance promotes the generation of ROS and impairs the distribution of histone H3 lysine 27 trimethylation (H3K27me3) at the CXCL1 promoter. Consequently, altered CXCL1 expression recruits MDSCs into the tumor microenvironment via CXCR2, thereby contributing to resistance to ICB therapy.^167^ CRCs also exhibit TDO2 upregulation, in turn activating the Kyn–AhR pathway, which increases glycolysis to drive the recruitment of M2-like TAMs via an AhR-dependent mechanism and promote CCL5 secretion.^168^ CD73–adenosine metabolism transcriptionally upregulates CCL5 through tumor cell-autocrine adenosine–Adora2a signaling-mediated activation of the p38–STAT1 axis, recruiting regulatory T (Treg) cells to form an immunosuppressive TME.^169^ Metabolically driven cytokine release serves as a key mechanism to shape immunosuppressive TME dynamics, highlighting metabolite‒cytokine axes as actionable targets to increase immunotherapy efficacy.

### Enemies or allies: immunological remodeling in the tumor microenvironment

Cancer cells can alter the metabolic state of immune cells through mechanisms such as competition for essential nutrients.^170^ (Fig. 3d) and the production of abnormal metabolic intermediates (Fig. 3c).^171^ This metabolic reprogramming reorients immune cytotoxic cells from an activated to a suppressed phenotype, which is characterized by the upregulation of immune tolerance-associated molecules, diminished production of cytotoxic molecules and cytokines and the amplification of inhibitory signaling pathways.^172^ As a result, an immunosuppressive TME is created by recruiting suppressive immune cells and preventing effective antitumor responses. Furthermore, the intercellular flow of metabolites further exacerbates alterations in the metabolic microenvironment.^173^ Therefore, the identification of key immunometabolic factors may provide insights into the complexity of the metabolic microenvironment.

#### Lactate and glucose metabolism

The TME is characterized by profound hypoxia and acidosis, which critically influence immune cell behavior via reduced levels of nutrients and oxygen and a buildup of lactic acid. In this hypoxic milieu, key metabolic regulators, such as LDHA,^174^ carbonic anhydrase 9 (CA9),^175^ and glucose transporters (GLUTs),^176^ modulate intra- and extracellular pH, thereby maintaining an acid-suppressive TME. CD8 + T cells and natural killer (NK) cells are critical mediators of antitumor immunity. However, a major product of LDHA impedes the activation of NFAT in T cells and NK cells, reducing their ability to produce IFN-γ.^177^ Moreover, HIF-mediated regulation of vascular endothelial growth factor-A (VEGF-A) in CD8 + T cells accelerates tumor progression and disrupts vascular patterns, further emphasizing the role of HIF in shaping the TME.^178^ Notably, CD8 + T cells differentiate into a dysfunctional state, known as T-cell exhaustion, after persistent TCR stimulation in the TME. Exhausted T (Tex) cells, characterized by the upregulation of coinhibitory molecules and reduced polyfunctionality, also overexpress monocarboxylate transporter 1 (MCT1), facilitating the increased uptake of lactic acid.^179^ In addition to its classical functions, LDHA produces L-2-hydroxyglutarate (L-2HG), an epigenetic modifier that induces histone hypermethylation, impairing T-cell proliferation and migration.^180^ LDHA also facilitates the PI3K-dependent inactivation of Foxo1, a crucial TF for Teff cell responses, thus further contributing to immune evasion.^171^ This clearly reflects an epigenetic activation mechanism mediated by metabolic enzymes (Fig. 2b).

CD4 + T cells, especially Treg cells, also experience functional and differential changes. Under hypoxic conditions, transforming growth factor-beta (TGF-β) synergizes with HIF to promote CD4 + T-cell differentiation into immunosuppressive Treg cells through direct interaction with the FOXP3 promoter.^181^ In addition, lactate alters Foxp3-dependent RNA splicing, preserving the phenotypic and functional stability of Treg cells via CTLA-4 signaling.^182^ Intriguingly, lactate induces PD-1 expression on both CD8 + T cells and Treg cells, influencing the efficacy of PD-1 blockade therapies by modulating the competition for reactivation between these cell populations. In tumors with high glycolytic activity, Treg cells preferentially absorb lactate through MCT1, promoting NFAT1 translocation to the nucleus and increasing PD-1 expression, whereas Teff cells exhibit reduced PD-1 levels.^183^ For specific epigenetic modification sites, H3K18la and H3K9la act as transcription initiators of key genes regulating T cell function.^184^ These findings reveal that lactate acts as a substrate for histone lactylation, initiating metabolic‒epigenetic integration (Fig. 2a).

In addition to its role in lymphoid cells, HIF-1α enhances the expression of VISTA on myeloid cells through its binding to a conserved hypoxia response element in the VISTA promoter, which leads to the suppression of T-cell activity.^185^ Lactate activates mTORC1 signaling, which in turn suppresses TFEB-mediated expression of the macrophage-specific vacuolar ATPase subunit ATP6V0d2, revealing the role of lactate in activating key metabolite-sensing receptors to induce immune cascades (Fig. 2). This cascade enhances the HIF-2α-driven production of VEGF and contributes to the development of a protumoral macrophage phenotype.^186^ Additionally, lactylation of retinoic acid-inducible gene 1 (RIG-1) suppressed the recruitment of NF-κB to the Nlrp3 promoter in macrophages, thereby affecting the immunosuppressive activities of Treg cells and the antitumor activities of CD8^+^ T cells.^187^ PERK-driven glucose metabolism promotes IL-10 expression and MDM immunosuppressive activity via histone lactylation.^38^ In addition to TAMs, lactylation at histone H3 lysine 18 (H3K18la) facilitates immunosuppression by increasing METTL3 expression, which, in turn, catalyzes the m6A modification of JAK1 mRNA in tumor-infiltrating myeloid cells, promoting STAT3 phosphorylation.^188^ In contrast, the loss of β2-integrin-mediated adhesion in bone marrow-derived DCs induces a suppressed metabolic state, characterized by reduced metabolic activity, diminished ROS production, and impaired glucose uptake. This metabolic reprogramming is accompanied by epigenetic changes that downregulate the expression of costimulatory markers (CD86, CD80, and CD40), cytokines (IL-12), and the chemokine receptor CCR7, ultimately impairing dendritic cell function.^189^ Therefore, glucose metabolism, especially glycolysis, serves as a central hub for rewiring myeloid cell immunoregulatory landscapes in the TME (Fig. 3e).

#### Tricarboxylic acid cycle intermediates

Metabolites derived from the Krebs cycle have been implicated in various signaling pathways, significantly influencing immune cell activation and tumorigenesis.^190^ Notably, succinate, itaconate, fumarate, and D2-hydroxyglutarate (D-2HG) play direct roles in immune modulation and cancer progression.^191^ Cancer cells release succinate into the TME, where it activates SUCNR1, initiating a PI3K-HIF-1α axis that polarizes macrophages into TAMs^192^ and suppresses the cGAS-interferon-β pathway, which limits CD8 + T cell trafficking to the TME.^193^ In addition, depletion of fumarate hydratase (FH) in cancer cells leads to fumarate accumulation in the interstitial fluid, disrupting ZAP70 activity,^194^ and activating the fumarate-DAPK1-mTORC1 pathway in infiltrating CD8 + T cells,^195^ resulting in suppressed CD8 + T cell activation and impaired antitumor immune responses. Oncogenic mutations in IDH produce D-2HG, which destabilizes HIF-1α, shifting metabolism toward oxidative phosphorylation. This alteration increases the frequency of Treg cells and reduces T helper 17 (Th17) polarization, further promoting immune evasion.^196^ In activated T cells, S-2-hydroxyglutarate (S-2HG) predominates over R-2-hydroxyglutarate and serves as an immunometabolite that induces epigenetic modifications, including elevated H3K27me3 levels and reduced 5hmC in CD8 + T cells.^197^ Lactate and intermediates of the TCA cycle exhibit reciprocal interactions that impact immune cell function instead of independent effects. Metabolically adaptive cytotoxic T cells utilize succinate as an autocrine signal through SUCNR1, a process that requires pyruvate carboxylase (PC) to replenish TCA cycle intermediates. However, lactate inhibits PC-mediated anaplerosis, impairing the cytotoxic potential of CD8 + T cells.^198^

A related metabolic shift in TAMs involves the upregulation of OXCT1, leading to increased succinate levels, which mitigates CD8 + T-cell exhaustion through the succinate-H3K4me3-Arg1 axis.^199^ Previous studies have also highlighted the role of PHGDH,^200^/PSAT1^201^-mediated serine biosynthesis and glutaminolysis in promoting α-ketoglutarate (αKG) production. αKG is essential for the activation of mTORC1 signaling and the maintenance of an M2-like macrophage phenotype, including the upregulation of PD-L1 expression in the TME. This process is facilitated through JMJD3-dependent histone modification.^200,201^ L2HGDH-mediated S-2HG catabolism orchestrates macrophage polarization to elicit antitumor immunity by increasing the accessibility of proinflammatory genes to chromatin.^202^ Furthermore, the upregulation of aconitate decarboxylase 1 (ACOD1) in TANs promotes the production of itaconate, a metabolite that activates Nrf2 signaling to confer resistance to ferroptosis, thereby supporting TAN persistence.^203^ Collectively, TCA cycle intermediates—succinate, fumarate, D-2HG, αKG, and itaconate—orchestrate myeloid (e.g., TAMs and TANs) and lymphoid (e.g., CD8 + T cells and Tregs) cell differentiation and function, which act as double-edged swords to promote antitumor immunity or fuel immune evasion.

#### Lipid metabolism

In the TME, fatty acids are potent immunosuppressive factors,^204^ and lipid accumulation is linked to increased expression of CD36, a receptor that mediates the uptake of oxidized low-density lipoproteins (OxLDLs) into T cells. This uptake induces lipid peroxidation and activates the p38 kinase pathway, contributing to T cell dysfunction. Indeed, CD36 is upregulated by both Teff cells and Treg cells exposed to fatty acid accumulation in the TME but mediates diametrically divergent effects. On the one hand, Teff cells respond to CD36-dependent fatty acid uptake by experiencing widespread oxidative stress coupled with impaired secretion of effector molecules such as IFNG, tumor necrosis factor (TNF), and perforin 1 (PRF1).^205,206^ On the other hand, CD36 favors the metabolic adaptation of intratumoral Treg cells to a lactate-enriched microenvironment, de facto promoting their immunosuppressive functions.^207^ In addition, interleukin-7 (IL-7) stimulates the release of HMGB1, which enhances CD8 + T cell proliferation and IFN-γ production through FAO.^208^ Moreover, ketogenesis-derived β-hydroxybutyrate, found in CD8 + T memory cells, induces epigenetic modifications, such as H3K9 β-hydroxybutyrylation of Foxo1 and PGC-1α, thereby rerouting carbon flux toward gluconeogenesis and the pentose phosphate pathway, which are essential for memory formation.^209^ Additionally, phospholipid metabolism mediated by Plpp1 in intratumoral CD8 + T cells is altered, and PLPP1 loss promotes CD8 + T cell ferroptosis and impairs antitumor immunity.^210^

In contrast, TAMs exhibit obviously heterogeneous metabolic profiles that are intricately linked to their functional roles within the TME.^211^ Lipid-loaded FABP5+ TAMs utilize long-chain unsaturated fatty acids (UFAs) released by cancer cells to activate peroxisome proliferator-activated receptor (PPAR), which in turn increases the expression of immune checkpoint ligands and immunosuppressive molecules.^212^ Similarly, C1q+ TAMs promote fatty acid metabolism via FABP5, activating PPARγ and upregulating genes associated with immune suppression.^213^ Additionally, the accumulation of 25-hydroxycholesterol (25HC) in the lysosomes of TAMs can compete with cholesterol for binding to GPR155, thereby inhibiting mTORC1 activity. This disruption activates AMPKα, which phosphorylates STAT6 at Ser564, enhancing STAT6 activation and subsequent production of the immunosuppressive enzyme arginase 1 (ARG1).^214^ Elevated expression of apolipoprotein E (ApoE) in TAMs modulates cholesterol metabolism and triggers the production of the chemokines CXCL1 and CXCL5 via LDL receptor and NF-κB signaling, further contributing to the immunosuppressive TME.^215^ In addition, tumor-associated neutrophils (TANs) of the N1 type, which are increasingly recognized for their potential to overcome immunosuppressive barriers, also exhibit metabolic adaptations that enhance responses to immunotherapy. A ketogenic diet has been shown to modulate TAN polarization via the AMOT-YAP/TAZ axis, thereby inhibiting CRC progression.^216^ To summarize, lipid metabolism critically bridges epigenetic and immune regulation by modulating histone modifications, initiating receptor signaling in immune cells—driving immunosuppression or enabling proimmunogenic adaptations—positioning lipid metabolic pathways as dual therapeutic targets to disrupt immune evasion or enhance antitumor immunity.

#### Amino acid and one-carbon metabolism

One-carbon metabolism (1CM), comprising folate metabolism and methionine metabolism, serves as an important mechanism for providing cellular energy and the production of vital signaling molecules, including single-carbon moieties.^217^ Its regulation is instrumental in sustaining the proliferation of cancer cells and T cell-mediated immunotherapy.^218^ Key enzymes in the 1CM pathway, such as serine hydroxy methyltransferase 2 (SHMT2), MTHFD2, and DNMT1, are closely associated with T cell immune function and influence the efficacy of T cell-based immunotherapy.^219^ Metabolites involved in the 1CM, such as methionine, formate, and SAM, could mediate the T cell immune response. Similarly, cancer cells exhibit aggressive methionine consumption, outcompeting T cells for this essential amino acid by overexpressing the methionine transporter SLC43A2 (Fig. 3d). Decreased intracellular methionine levels, along with decreased availability of the methyl donor (SAM), result in reduced dimethylation of histone H3 at lysine 79 (H3K79me2), which suppresses STAT5 expression and impairs T-cell immunity.^220^ Oral formate augments the fitness of CD8 + T cells within the TME, promotes tumor clearance^221^ and promotes the survival of Foxp3+ Treg cells,^222^ which function in intestinal immunity. Additionally, folate receptor 4 (FR4), a subtype of the receptor of folic acid, is expressed at high levels on Treg cells,^223^ and a Treg cell-depleting anti-FR4 antibody helps to enhance antitumor responses in a poorly immunogenic melanoma model.^224^ These studies emphasized the key role of 1CM in regulating immunity by providing SAM.

Cancer cells can outcompete CD8 + T cells for taurine by overexpressing the taurine transporter SLC6A6, leading to taurine depletion in T cells, which induces cell death and dysfunction, thereby promoting tumor progression. Mechanistically, taurine deficiency in CD8 + T cells results in increased endoplasmic reticulum (ER) stress, triggering ATF4 transcription through a PERK-JAK1-STAT3 signaling-dependent pathway. Elevated ATF4 then transactivates multiple immune checkpoint genes, contributing to T cell exhaustion.^170^ In addition, low levels of arginine activate T cells to undergo metabolic and transcriptional reprogramming via the ATF4-SLC7A11-GSH axis, which helps to preserve their Treg cell-like suppressive function.^220^ In contrast, glutamate cysteine ligase (Gclc)-mutant Treg cells rely on increased serine uptake and synthesis to compensate for reduced glutathione (GSH) levels and activate mTORC1, although this reprogramming downregulates FoxP3 expression and limits the immunosuppressive function of Treg cells.^225^ In addition to lymphoid cells, the metabolic and epigenetic reprogramming of myeloid cells often results in a protumor phenotype rather than an antitumor phenotype; these cells are primary competitors rather than providers of glucose^226^ and arginine,^227^ which are critical for T-cell expansion.^228^ Additionally, serine deprivation reduces IL-1β production by inhibiting mTOR signaling and reprogramming the transcriptomic and metabolic profiles of M1 macrophages.^229^ In summary, amino acid metabolism critically shapes immune‒epigenetic crosstalk, especially in the TME, where cancer cell-driven depletion of taurine, arginine, or serine reprograms T cell exhaustion, Treg immunosuppression and myeloid cell polarization. While these pathways highlight amino acids as dual regulators of epigenetic states and immune dysfunction, deeper interrogation to identify therapeutic strategies targeting amino acid metabolism in cancer immunology is necessary.

#### Ionic signals

Ionic signals play a critical role in modulating adaptive antitumor immune responses.^230^ Tumors often contain regions of cellular necrosis, which are associated with poor patient prognosis across various cancer types.^231^ These necrotic areas release intracellular potassium ions into the TME, leading to an increase in the extracellular potassium concentration, which impairs TCR-driven Akt–mTOR signaling and the activation of effector programs to limit T cell functionality^232^ (Fig. 3c). This elevation also restricts nutrient uptake, triggering autophagy and reducing histone acetylation at key loci related to T cell effector function and exhaustion, which in turn results in improved in vivo persistence and multipotency of CD8 + T cells.^233^ In contrast, sodium chloride (NaCl) enhances the activation and effector functions of human CD8 + T cells, a process linked to improved metabolic fitness. Mechanistically, NaCl-induced changes are mediated by the increase in Na + /K + -ATPase activity, which leads to membrane hyperpolarization. This, in turn, amplifies the electromotive force for TCR-induced calcium influx, enhancing downstream TCR signaling and effector responses.^234^ While potassium and sodium ions critically modulate T cell functionality, the roles of other ions (e.g., calcium, magnesium, chloride) in shaping immune dynamics remain underexplored, underscoring the need to dissect ionic diversity in the TME as a novel frontier for cancer immunotherapy.

#### Metabolites from microbiome

The tumor and gut microbiomes play crucial roles in modulating immune responses by influencing the complex metabolic microenvironment (Fig. 3f). The metabolic pathways of the gut microbiome interact dynamically with host gene products through various bioactive molecules, particularly intestinal bile acids (BAs). These BAs serve as critical hormones that regulate cholesterol metabolism and energy homeostasis by binding to nuclear and G protein-coupled receptors.^235^ These receptors are critical for shaping innate immune responses and have been shown to increase the frequency of colonic RORγ+ Treg cells^236^ while simultaneously reducing Ca2 + -NFAT2 signaling by enhancing PMCA activity. Deoxycholic acid, a byproduct of BA metabolism, further suppresses antitumor CD8 + T cell responses.^237^ D-lactate, another metabolite produced by the gut microbiome, acts as an endogenous immunomodulator, promoting the clearance of pathogens by Kupffer cells and facilitating the conversion of M2 TAMs to the more proinflammatory M1 phenotype.^238^

Within the tumor microbiome, Lactobacillus plantarum L168 and its metabolite, indole-3-lactic acid, enhance dendritic cell function by increasing histone acetylation at the IL12a enhancer region, thereby increasing IL-12a production. This process primes CD8 + T cells for more effective antitumor immunity.^239^ Furthermore, indole-3-lactic acid modulates the cholesterol metabolism of CD8 + T cells by altering chromatin accessibility, leading to enhanced T cell function and improved responses against tumor growth.^239^ Additionally, supplementation with Lactobacillus johnsonii or the tryptophan-derived metabolite indole-3-propionic acid (IPA) has been shown to increase the efficacy of anti-PD-1 immunotherapy. IPA, produced in synergy with Clostridium sporogenes, influences the stemness and exhaustion profiles of CD8 + T cells by promoting H3K27 acetylation (H3K27ac) at the superenhancer region of Tcf7, thereby facilitating the generation of progenitor exhausted CD8 + T cells (Tpex).^240^ Butyrate-producing bacteria, such as Roseburia, are enriched in lung cancer patients with early recurrence. Intratumoral butyrate production inhibits HDAC2 expression, upregulates H19, and promotes M2 macrophage polarization, thereby increasing metastasis.^241^ Furthermore, the activity of the macrophage aryl hydrocarbon receptor (AhR), which is crucial for immune modulation, depends on the metabolism of dietary tryptophan by Lactobacillus. TAMs exhibit elevated AhR activity, and in the absence of AhR, macrophages adopt a more inflammatory phenotype with a reduction in TNFα + IFNγ + CD8 + T cells.^242^ Thus, the gut and intratumoral microbiomes collectively form a tumor-associated microbial ecosystem that orchestrates metabolic-epigenetic-immune cascades by producing bioactive metabolites (Fig. 3f).

## Multilevel regulatory mechanisms of the metabolic-epigenetic-immune axis in cancer progression

**Fig. 4:**
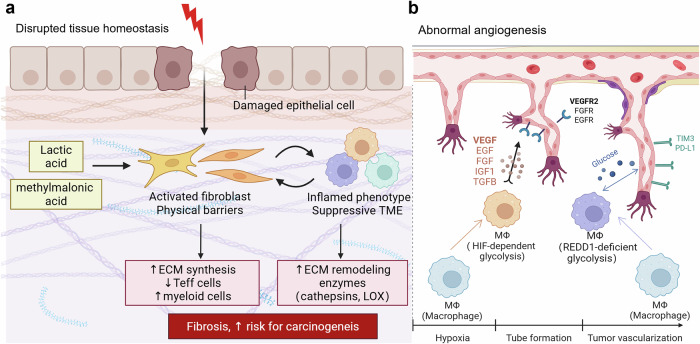
The role of the metabolic-epigenetic-immune axis in tumorigenesis. Tumorigenesis disrupts tissue homeostasis and immune equilibrium, with the formation of neovasculature being crucial for providing essential nutrients to the tumor. Local tissue metabolic alterations can trigger signaling pathways in fibroblasts and macrophages, leading to extracellular matrix remodeling and promoting angiogenesis. **a** Extracellular matrix remodeling involves the reorganization of structural components that facilitate tumor progression. **b** Angiogenesis ensures a continuous supply of blood vessels to nourish the growing tumor. Both processes contribute to the development of a locally immunosuppressive TME, which further enhances carcinogenesis. The figure was generated with BioRender (https://biorender.com). TME tumor microenvironment. TME tumor microenvironment, ECM extracellular matrix

**Fig. 5:**
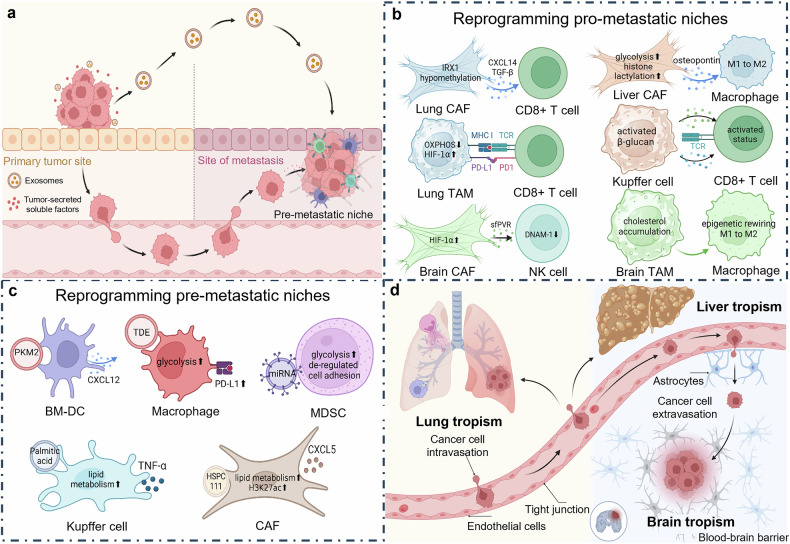
Bidirectional selection and coadaptation mechanisms between tumor cells and target organs. The process by which cancer cells adapt to target organs can be divided into two distinct mechanisms: remote regulation and direct adaptation. Remote regulation primarily influences stromal and myeloid cells, which exhibit commonalities across various types of tumors (**a**, **c**). In contrast, direct adaptation within specific organs is characterized by organ-specific responses, with the liver, lungs, and brain being the most prominent sites of such interactions (**b**, **d**). The figure was generated with BioRender (https://biorender.com). BM-DCs bone marrow-derived dendritic cells, CAFs cancer-associated fibroblasts, TAMs tumor-associated macrophages, DCs dendritic cells, MDSCs myeloid-derived suppressor cells

**Fig. 6:**
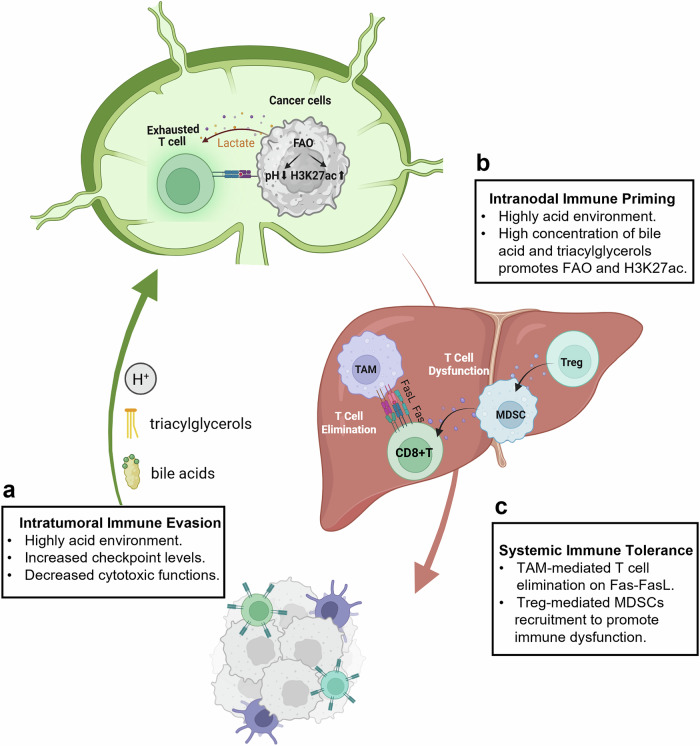
The role of lymph nodes and the liver in systemic immune tolerance during tumor progression. In addition to primary tumor site (**a**), the lymph nodes (**b**) and liver (**c**) are critical organs involved in the induction of immune tolerance. These organs are intricately linked through both the blood and the lymphatic circulation, where they cooperate to orchestrate the systemic immune suppression associated with tumor progression. Primary tumors establish an acidic microenvironment, accompanied by enhanced immune checkpoint expression and suppression of immune cell function to achieve immune escape (**a**). Metabolites such as lactate, bile acids, and triglycerides generated within this milieu can reach tdLNs via lymphatic circulation, inducing T-cell exhaustion (**b**). Lymphocytes entering the liver through the bloodstream similarly undergo immunosuppression, predominantly mediated by interactions with TAMs and MDSCs (**c**). The figure was generated with BioRender (https://biorender.com). *tdLNs* tumor-draining lymph nodes, *TAM* tumor-associated macrophage, *Treg* regulatory T cell, *MDSC* myeloid-derived suppressor cell

Cancer cells embark on a complex and multifaceted journey, utilizing a combination of metabolic, epigenetic, and immune-modulatory strategies to adapt, proliferate, and colonize distant organs. This relentless drive mirrors the exploration of vast and hostile territories within the human body. In the initial phase of this voyage, cancer cells demonstrate remarkable self-sustaining mechanisms, allowing them to survive and thrive in hostile microenvironments. Simultaneously, a profound transformation occurs within the TME: the immune system, once a “vigilant defender”, is either coopted or evaded by cancer cells, as shown in Fig. 2. During carcinogenesis, it stands for the beginning of the journey into darkness (Fig. 3). This intricate interplay extends beyond the confines of the primary tumor, where the “Lorelei signal” emerges, guiding metastatic cells to distant organs—fertile new sites for colonization (Fig. 4). Certainly, cancer cells are subjected to various external attacks, such as immune therapies, which promote further adaptation to ensure their survival—illustrating the phenomenon of treatment resistance. As the journey continues, cancer cells orchestrate systemic reprogramming of the immune landscape, akin to explorers adapting their defenses to meet evolving challenges (Fig. 5). By unraveling the complex forces driving this “cosmic” battle (Fig. 6), we may chart a course for more effective therapies, advancing beyond the limitations of current treatment paradigms.

### Regulation of tumorigenesis

#### Into darkness: subverted adaptive-innate cell crosstalk and tissue homeostasis

Malignant cells must overcome several critical bottlenecks to successfully establish a tumor, namely, carcinogenesis. These include evading immune surveillance, converting the surrounding stroma into a tumor-supportive microenvironment, and ensuring an adequate supply of oxygen and nutrients to sustain high metabolic demands^243^ (Fig. 4). Notably, the recruitment and activation of myeloid cells, particularly macrophages^244^ and neutrophils,^245^ play pivotal roles in this process. These cells often polarize toward an immunosuppressive phenotype and secrete ROS, proinflammatory cytokines, chemokines, growth factors, and proangiogenic factors, all of which contribute to the creation of an inflammatory microenvironment conducive to tumor development.^246^ This inflammatory milieu facilitates tissue damage, epithelial mutagenesis, angiogenesis, immunosuppression, and extracellular matrix remodeling, collectively promoting tumor initiation.^247^

First, regardless of whether cancer arises from prolonged chronic inflammation^248^ or precancerous lesions^249^ during early tumorigenesis, virtually all advancing tumors elicit varying degrees of exclusion or dysfunction in T cells,^250^ NK cells,^251^ and DC exclusion.^252^ Myeloid cells utilize various mechanisms to shift the balance from immune activation to immune evasion.^253^ Pan-cancer single-cell RNA sequencing (scRNA-seq) analyses have identified a distinct subset of TAMs characterized by the expression of IL-4I1, PD-L1, and IDO1, which is associated with T-cell exhaustion and the accumulation of Treg cells.^254^ IDO1 and IL-4I1 are recognized as key regulators of AhR activation, functioning as metabolic immune checkpoints that promote tumor progression through tryptophan catabolism.^255^ Additionally, polymorphonuclear MDSCs, which are pathologically activated neutrophils, play a pivotal role in mediating immune suppression through the uptake of arachidonic acid via fatty acid transport protein 2 (FATP2) and the subsequent synthesis of prostaglandin E2.^256^ Furthermore, myeloid cells contribute to immune evasion by depleting cystine and cysteine, which are essential for T cell activation.^257^

In addition to immune suppression, extracellular matrix (ECM) remodeling is a critical driver in establishing a tumor-promoting microenvironment.^258^ Cancer-associated fibroblasts (CAFs) are activated through various mechanisms, including metabolic alterations that foster a fibrotic landscape.^259^ In hypermetabolic areas of the TME, fibroblasts utilize lactate produced by glycolysis to differentiate into inflammatory CAFs (iCAFs).^260^ In addition, the oncometabolite methylmalonic acid (MMA), which accumulates with aging, has been implicated in the activation of fibroblasts within the TME.^261^ Once activated, CAFs are central to the development of fibrotic tumors, which often exhibit an inflamed phenotype populated by myeloid cells. These myeloid cells are key sources of ECM remodeling enzymes, including matrix metalloproteinases (MMPs), cathepsins, and collagen-crosslinking enzymes such as lysyl oxidase (LOX).^262^ These enzymes drive fibrosis and perpetuate a positive feedback loop that reinforces the fibrotic microenvironment. Moreover, this fibrotic milieu can directly hinder T cell recruitment and activation, as CAFs release chemokines such as CXCL12^259^ and cytokines such as TGF-β^260^ and facilitate the formation of physical barriers through ECM deposition.^263^ In short, metabolic remodeling activates CAFs into iCAFs, which can recruit myeloid cells to secrete ECM-remodeling enzymes and release inflammatory mediators. This establishes a self-reinforcing profibrotic and proinflammatory feedback loop that exacerbates immunosuppression and accelerates carcinogenesis (Fig. 4a).

Angiogenesis, the formation of new blood vessels, is a critical process in tumorigenesis.^264^ Once a tumor exceeds 1–2 mm in size, it must develop its own vascular supply to provide essential oxygen and nutrients.^265^ Hypoxia, or oxygen deprivation, is a primary driver of angiogenesis.^264^ Key molecules that respond to hypoxia facilitate angiogenic switching, with vascular endothelial growth factor (VEGF) and its downstream signaling pathways being the central mediators.^266^ In the TME, macrophages play a pivotal role in promoting angiogenesis by secreting proangiogenic factors and assisting in the degradation of the perivascular extracellular matrix.^267^ Hypoxic conditions trigger metabolic reprogramming in TAMs, leading to the transcription of proangiogenic genes.^268^ Specifically, hypoxic TAMs exhibit increased expression of HIF-dependent glycolytic genes, suggesting a preference for glycolytic metabolism.^269^ In addition, TAMs deficient in REDD1, which exhibit increased glycolytic activity, outcompete endothelial cells for glucose, thus preventing excessive vascular activation and promoting the formation of stable vascular junctions.^270^ Neutrophils and immature myeloid cells also play significant roles in the early stages of angiogenesis, as validated in experimental tumor models.^267^ Importantly, the relationship between the immune system and the vasculature is bidirectional. Tumor-induced angiogenesis not only supports tumor growth but also contributes to immune evasion. For example, the tumor vasculature can downregulate vascular adhesion molecules such as ICAM-1, VCAM-1, E-selectin, and P-selectin, which are crucial for immune cell trafficking.^271^ Conversely, immune checkpoint molecules, including IDO, TIM3, and PD-L1, can be upregulated on endothelial cells, facilitating immune suppression.^271^ Overall, the glycolytic reprogramming of macrophages fuels angiogenesis while simultaneously fostering immune suppression through endothelial checkpoint upregulation and impaired immune cell trafficking, illustrating how TAM plasticity connects vascular remodeling and immune evasion to sustain carcinogenesis (Fig. 4b).

### Invasion and metastasis

#### Lorelei Signaling: Remote Regulation of the Premetastatic Microenvironment

The primary tumor secretes proteolytic enzymes, chemokines, and extracellular vesicles (EVPs) that systematically reprogram host tissue, establishing a premetastatic microenvironment characterized by vascular hyperpermeability, immunosuppressive cell infiltration, and extracellular matrix remodeling—a permissive niche preceding metastatic colonization.^272^ EVPs play a crucial role in the formation of premetastatic niches, reprogramming cell functions across distant, metastasis-free organs^273^ (Fig. 5a). Notably, the liver has been identified as a primary target of tumor-derived EVPs.^274^ Furthermore, exosomes containing miR-122 can interact with astrocytes and neurons, thereby influencing the metabolic microenvironment of the brain’s premetastatic niche.^275^ Additionally, tumoral exosomes have been shown to reprogram and activate fibroblasts.^276^ Tumor-derived miR-122 suppresses glucose uptake in the premetastatic niches of the brain and lungs by downregulating pyruvate kinase (PKM), thereby altering the glucose metabolism of recipient cells and contributing to the preparation of these organs for metastatic colonization.^277^

In the context of cancer progression, tumor-derived EVPs, such as exosomes, can induce significant metabolic changes in myeloid cells, facilitating the establishment of an immune-suppressive environment (Fig. 5c). Notably, tumor-derived exosomes facilitate the activation and expansion of MDSCs via intercellular communication, thereby contributing to the immunosuppressive TME.^278^ Moreover, the fatty acid cargo of tumor EVPs, particularly palmitic acid, has been shown to stimulate Kupffer cells to secrete TNF, creating a proinflammatory microenvironment in the liver.^279^ Tumor-derived exosomes also promote the polarization of macrophages toward a glycolytic-dominant phenotype. This occurs through the activation of Toll-like receptor 2 (TLR2) and NF-κB signaling, while lactate produced by cancer cells further feeds back on NF-κB to increase PD-L1 expression, ultimately reinforcing the immunosuppressive profile of macrophages.^280^ Stromal cells also play essential roles in the premetastatic TME. For example, CRC-derived exosomes containing HSPC111 can alter the lipid metabolism of CAFs by phosphorylating ATP-citrate lyase (ACLY), which increases acetyl-CoA production. This, in turn, increases the expression of CXCL5, initiating a CXCL5-CXCR2 signaling axis in CAFs and enhancing H3K27ac, which further supports the premetastatic niche.^281^ Prostate cancer-derived exosomes transfer PKM2 to bone marrow stromal cells (BMSCs), leading to increased CXCL12 production via a HIF-1α-dependent pathway.^282^ In addition to exosomes, early liver infiltration of myeloid cells mediates IL6–pSTAT3 immune–hepatocyte cross-talk, depleting a master metabolic regulator—HNF4α. This depletion disrupts glutamate and aspartate metabolism, triggering CCL2-mediated immune cell recruitment and further altering the metabolic landscape of the liver.^208^ These studies provide deep insights into metabolic preadaptation and immunosuppressive niche formation in the liver, yet similar mechanisms in other highly metastatic organs (e.g., the lungs and brain) remain underexplored. Targeting the disruption of premetastatic niche formation—through metabolic‒immune crosstalk or exosome-mediated pathways—could effectively terminate metastatic progression and improve therapeutic outcomes.

#### The Strange New World: Cellular reactions in target organs to remodel the TME

The survival and proliferation of cancer cells within specific organs are dictated by the outcome of complex and reciprocal interactions between cancer cells and various local resident and recruited cell populations, including bone marrow-derived immune cells.^283^ These interactions are shaped by both metabolic symbiosis—manifested as synergistic and antagonistic effects—and the balance of these opposing forces determines whether cancer cells will die, proliferate, colonize a new site, or enter a dormant state.^284^ Upon reaching a target organ, circulating cancer cells encounter a range of common cellular populations, such as CAFs, TAMs, and dendritic cells, as well as organ-specific cell types. For example, in the liver, cell interactions are focused mainly on sinusoidal endothelium, stellate cells, Kupffer cells, and portal fibroblasts; in the brain, they engage with astrocytes and neurons; and in the lungs, alveolar and interstitial macrophages are key players. These interactions are mediated through cell–cell and cell–extracellular matrix adhesion, as well as the release of soluble factors, which together influence the metastatic niche and impact tumor progression.^285^ Both the metastatic tumor and the primary tumor at the metastatic site may employ analogous adaptive strategies. For example, the gut microbiome utilizes BAs as messengers to mediate the chemokine-driven accumulation of hepatic natural killer T (NKT) cells through the CXCL16 level of liver sinusoidal endothelial cells. This interaction enhances antitumor immunity within the liver, providing protection against both primary and metastatic liver tumors.^286^ Similarly, neuronal activity can also promote the malignant behavior of cancer cells in both primary and metastatic brain tumors. Tumor cells do not disrupt the neuronal synapse but rather appear to adopt a position that would normally be occupied by astrocytic processes, allowing the neurons to release glutamate to activate themselves and promote their invasion and growth. Mechanically, astrocyte-induced mGluR1-expressing cancer cells become overly dependent on glutamate signaling through epidermal growth factor receptor (EGFR) stabilization, which is advantageous for adaptation to the brain microenvironment.^287^ However, the receptors on glioma cells that respond to glutamate stimulation are AMPA receptors,^288^ which differ from NMDA in brain metastatic breast cancer.^289^ This finding illustrates that metastatic and primary tumors within the same organ exhibit both conserved and specific strategies for adapting to local microenvironments, which underscores the importance of comparative studies to dissect their shared mechanisms and divergent pathways to optimize therapeutic interventions.

Cells common to multiple organs, including fibroblasts,^290^ and macrophages,^291^ often exhibit distinct characteristics in response to different environments. Recent advancements in scRNA-seq have revealed the considerable heterogeneity and multifaceted tumor-supporting functions of CAFs.^290^ Distinct CAF phenotypes have been shown to impede T cell access and cytotoxicity through a variety of mechanisms (Fig. 5b). Specifically, myCAFs promote the synthesis of ECM components, thereby creating a physical barrier that restricts T cell infiltration.^292^ CAFs expressing major histocompatibility complex II (MHC-II) molecules facilitate antigen presentation and TCR engagement, resulting in T cell dysfunction.^293^ Additionally, iron-loaded CAFs, termed FerroCAFs, activate the iron-dependent epigenetic enzyme KDM6B, which induces an accessible chromatin state and drives the transcription of myeloid cell-associated genes such as CCL2, CSF1, and CXCL1, thus recruiting immunosuppressive myeloid cells to the TME.^294^ Tissue organ-specific CAFs also exhibit unique mechanisms of immune modulation. In brain metastasis, hypoxia-induced stabilization of HIF-1α upregulates fucosyltransferase 11, which fucosylates PVR and promotes its secretion from brain metastatic CAFs (bmCAFs).^295^ This process inhibits DNAM-1-mediated antitumor responses by NK cells.^296^ In liver metastasis of PDAC, myCAF-derived osteopontin (*Spp1*) supports immunosuppressive macrophage functions in a STAT3-dependent manner.^297^ Notably, IRX1-induced DNA hypomethylation enhances the production of TGF-β by lung myCAFs in response to CXCL14, thereby dampening CD8 + T-cell-mediated antitumor immunity and promoting lung metastasis.^298^ Advances in scRNA-seq have revealed the extensive heterogeneity of CAFs, facilitating an understanding of organ-specific adaptive strategies—such as ECM remodeling by myCAFs, MHC-II-mediated T cell dysfunction, and FerroCAF-driven myeloid recruitment—which collectively shape immunosuppressive niches. This functional plasticity enables CAFs to tailor immune evasion mechanisms to distinct metastatic microenvironments (e.g., the brain, liver, and lung), highlighting their pivotal role in orchestrating tissue-specific tumor-stroma crosstalk and metastasis progression.

Furthermore, stellate cells are a major source of CAFs in the liver (hepatic stellate cells, HSCs) and pancreas (pancreatic stellate cells, PSCs). TGF-β-induced activation of HSCs increases the expression of *GLUT1* to increase glucose uptake and glycolysis. This metabolic shift increases the secretion of tumor-promoting factors, such as Wnt, FGF, interleukins, and sphingosine-1-phosphate (S1P), which may modulate immune cell functions and contribute to liver metastasis in CRC.^299^ Additionally, histone lactylation in HSCs can reprogram cell‒cell interactions, influencing immune responses through the target NPIPB3.^300^ HSCs also secrete exosomes containing hexokinase 1 (HK1) to promote glycolysis and the creation of an acidic TME,^301^ initiating a metabolic-epigenetic-immune signaling network. Intriguingly, PSC-derived exosomes carry metabolites such as amino acids, acetate, stearate, palmitate, and lactate, which outcompete glucose- and glutamine-derived carbon to fuel the TCA cycle. This phenomenon supports nonessential amino acid and lipid biosynthesis, thereby remodeling the TME in a manner reminiscent of micropinocytosis.^302^ However, CAFs in PDAC with altered metabolism, particularly those with elevated IDO1 and arginase (ARG1, ARG2) levels, deplete critical amino acids such as tryptophan and arginine, which are essential for the proliferation and activation of Teff cells, further impairing antitumor immunity.^303^ Taken together, PSCs play a pivotal role in modulating the TME, contributing to the hypoxic, acidic conditions that limit cytotoxic T cell trafficking, promote macrophage differentiation into the protumor M2 phenotype, and recruit MDSCs and tumor-associated neutrophils to the tumor site.^304^

Additionally, macrophages in different tissues exhibit distinct metabolic and epigenetic profiles that contribute to tumor progression^291^ (Fig. 5b). In the brain, tumor-associated astrocytes influence cholesterol efflux via the ATP-binding cassette transporter (ABCA1), which upregulates the expression of chemokines such as CCL2 and CSF1, which in turn orchestrates the recruitment of TAMs.^300^ Furthermore, the phagocytosis of cholesterol-enriched myelin debris by TAMs results in the acquisition of a lipid-laden phenotype, which reflects cholesterol accumulation, epigenetic reprogramming, and the acquisition of immunosuppressive traits.^305^ Similarly, respiratory viral infections can induce reprogramming of mucosal-resident alveolar macrophages, enhancing their phagocytic capacity and cancer cell cytotoxicity. This reprogramming fosters long-lasting and tissue-specific antitumor immunity, which is linked to metabolic, transcriptional, and epigenetic resistance to tumor-induced immune suppression.^306^ Moreover, chronic exposure to airborne carbon black ultrafine particles—produced from incomplete organic combustion—result in selective mitochondrial damage in lung macrophages. This damage impairs oxidative respiration, sustains activation of the HIF-1α axis, and promotes glycolysis and lactate production, which initiates a lactate-driven epigenetic immune evasion cascade characterized by diminished Teff cell activation, expansion of Treg cells, and upregulation of PD-L1 + PD-L2 + CD206+ dendritic cells/macrophages. Furthermore, this cascade recruits inflammatory Ly6C+ monocytes and interstitial macrophages from the bone marrow, amplifying immunosuppression, as alveolar macrophages lose their ability to regulate inflammation.^307^ In contrast, the activation of liver-resident macrophages (Kupffer cells) by β-glucan inhibits cancer cell proliferation and promotes productive T cell-mediated responses against liver metastasis.^308^ Macrophages exhibit highly tissue-specific differentiation marked by functional duality (pro- vs. antitumor) and subpopulation diversity—shaped by organ-specific metabolic and epigenetic reprogramming—as evidenced by cholesterol-laden immunosuppressive TAMs in the brain, glycolysis-driven PD-L1+ lung macrophages, and β-glucan-activated Kupffer cells in the liver. This plasticity enables macrophages to adopt context-dependent roles in organ-specific immune dynamics (Fig. 5d).

### Resistance to immunotherapy

#### Survivors: Compensatory cytoprotective response to immune cytotoxicity

Immunotherapy, which takes advantage of the immune system to eliminate cancer cells, has been widely studied and applied in oncology. Despite the promising clinical outcomes of current cancer immunotherapies, the majority of patients either fail to respond to or develop resistance to ICB therapy.^309^ A comprehensive understanding of the mechanisms underlying immune resistance within the TME is crucial for identifying novel therapeutic targets and enhancing the efficacy of immunotherapies.^310^ The ‘Three Es Hypothesis’ describes immune cells and tumor cells waging battle on a continuum of elimination, equilibrium, and escape. Both tumor cell–intrinsic factors and tumor cell–extrinsic factors influence this balance.^311^ Notably, tumors often exploit metabolic adaptation strategies to circumvent immune-mediated cytotoxicity^312^ and withstand the immune stress induced by immunotherapy.^313^ These strategies frequently involve the inhibition of cell death signaling pathways or the compensatory activation of self-protective mechanisms.

In cancer immunotherapy, cellular cytotoxicity is actually a form of immunogenic cell death.^314^ CD8 + T cells are instrumental in tumor cell destruction, primarily through the activation of the FAS death receptor pathway and granzyme-mediated apoptosis or pyroptosis.^315^ Preclinical studies have demonstrated that glutamine metabolism inhibits FAS-mediated apoptosis by activating the NF-κB pathway in CRC and lung carcinoma models.^316^ In addition, ferroptosis—an iron-dependent form of cell death—has emerged as a key player central to the cytotoxic effects of Teff cells and the efficacy of ICB therapies.^317^ Ferroptosis is a distinct form of cell death characterized by iron accumulation, ROS and lipid peroxidation of cellular membranes.^318^ Its regulation is closely intertwined with fatty acid metabolism, including short-chain fatty acid (SCFA), medium-chain fatty acid (MCFA), long-chain fatty acid (LCFA), and very-long-chain fatty acid (VLCFA) metabolism,^319^ glucose metabolism^320,321^ and amino acid metabolism.^322,323^ Both arachidonic acid and IFNγ contribute to ferroptosis induction in cancer cells via the enzyme ACSL4.^319^ Lactate dehydrogenase (LDH) B, a subunit of active LDH with a known function in glycolysis, modulates GSH metabolism by regulating STAT1-dependent *SLC7A11* expression to promote ferroptosis defense in *KRAS*-driven lung cancer. In contrast, LDHB suppression promoted hyperactivation of glutamine metabolism, OXPHOS and mitoROS-dependent ferroptosis.^321^ SLC13A3, which mediates the uptake of itaconate in tumor cells, plays a crucial role in conferring resistance to ferroptosis by activating the NRF2-SLC7A11 pathway.^320^ Additionally, IFN-γ secretion significantly downregulates the expression of *SLC3A2* and *SLC7A11* in cancer cells, thereby reducing cystine uptake and promoting lipid peroxidation, which triggers ferroptosis.^322^ Additionally, Gln restriction or treatment with the Gln antagonist 6-diazo-5-oxo-L-norleucine leads to ferroptosis program activation in PDAC by mediating H3K4me3 upregulation and further transcriptional activation of *HMOX1* and *GPX4*.^323^ Furthermore, IL-1β-induced NNT acetylation enhances NADPH production, a process critical for maintaining iron‒sulfur clusters, thereby contributing to resistance to cancer immunotherapy.^324^ Collectively, resistance to ferroptosis—driven by metabolic rewiring (e.g., fatty acid, glucose, and glutamine metabolism)—constitutes a critical mechanism underlying immunotherapy resistance, highlighting ferroptosis modulation as a therapeutic imperative to overcome immune evasion.

Notably, autophagy, a conserved cellular response, is involved in preventing excessive immune activation,^325^ averting cell death, and maintaining homeostasis.^326^ Under hypoxic conditions, cancer cells across various histological types enhance resistance to immune effector molecules through autophagy activation.^327^ Specifically, tumor cells engage internal autophagic pathways to mitigate ROS accumulation, thus attenuating the antitumor efficacy of the STING pathway.^328^ In PDAC, autophagy promotes immune evasion by decreasing surface levels of MHC-I, thereby impairing antigen presentation.^329^ Additionally, IFN-α-induced TRIM14 transcription suppresses antitumor immunity through the recruitment of USP14, which inhibits the autophagic degradation of PD-L1.^330^ Autophagy in cancer plays a complex and dual role. While it typically supports tumor survival, evidence suggests that autophagy inhibition—such as through downregulation of BECN1 (encoding Beclin 1)—can promote tumorigenesis in breast cancer.^331^ In addition, increased expression of the autophagic marker microtubule-associated protein 1 light chain 3 beta (MAP1LC3B, or LC3B) is correlated with increased immune infiltration and improved patient outcomes in patients with breast carcinoma.^332^ Furthermore, autophagy has been shown to facilitate the degradation of tenascin C, an extracellular matrix protein involved in immune evasion, thereby promoting immune-targeted responses.^333^ The precise mechanisms underlying the role of autophagy in modulating immune therapy responses warrant further investigation.

The mechanism of autophagy activation in cancer cells is intricately linked to the TME, where cells utilize autophagy as a crucial survival strategy to cope with various stressors, such as nutrient deprivation.^334^ By harnessing autophagy-dependent catabolites—amino acids, fatty acids, nucleotides, and carbohydrates—tumor cells fuel biosynthetic processes and energy production.^335^ Under conditions of lipid scarcity, autophagy is further promoted by ADSL, which facilitates tumor progression through the fumarate-mediated inhibition of lysine demethylase 8 (KDM8).^336^ Additionally, chromatin remodelers and histone variants are implicated in the regulation of autophagy in response to nutrient fluctuations. For example, nitrogen starvation inactivates TORC1, which, in turn, triggers autophagy induction through repression of the Rpd3L complex, which deacetylates Ino80 and H2A. Z to suppress autophagy.^337^ In summary, understanding the metabolic‒epigenetic interplay driving autophagy initiation is crucial for disrupting autophagic survival pathways in cancer by targeting these mechanisms (e.g., inhibiting catabolite recycling or chromatin remodelers).

It has been proposed that a minority of patients experience hyperprogression (HP) following ICB therapy, characterized by an accelerated rate of tumor growth.^338^ Despite this observation, no significant histopathological or molecular markers reliably predict HP in advance, with rare exceptions such as MDM2 amplification and EGFR mutations.^339^ MDM2 and MDM4 act to inhibit the P53 transactivation domain and promote P53 degradation via proteasomal ubiquitination.^340^ Loss of P53 function is a well-known driver of oncogenesis, suggesting that the activation of tumor stemness may contribute to the development of HP. Intriguingly, studies have shown that patients with HP and those who achieve complete response (CR) after ICB therapy exhibit similar levels of tumor-infiltrating CD8 + T cells and an IFN-γ gene signature.^341^ First, IFN-γ has been shown to upregulate MDM2/4, further impairing P53 activity.^342,343^ In addition, patients with HP but not CR exhibit elevated expression of FGF2 and β-catenin signaling in tumors.^341^ Mechanistically, CD8 + T cell-derived IFN-γ targets FGF2 to selectively inhibit PKM2, a key enzyme in glycolysis, leading to reduced NAD+ production and increased β-catenin activity. This, in turn, fosters tumor stemness and increases tumorigenic potential.^341^ Thus, HP is driven by a complex interplay among metabolic, immunogenic, and oncogenic pathways.

The role of the immune microenvironment in the HP following ICB is gaining increasing attention, particularly with respect to the influence of innate immune cells on therapeutic responses.^344,345^ PD-1 receptors on innate immune cells—such as NK cells,^346^ dendritic cells,^345^ and monocytes —are pivotal in regulating immune responses. When these cells are exposed to anti-PD-1 antibodies or are PD-1 deficient, their effector functions, including the production of perforins, granzymes,^346^ and immunosuppressive cytokines such as IL-10, can be impaired, potentially exacerbating immunosuppression.^345^ This phenomenon is of particular concern in the context of HP, where PD-1 blockade may inadvertently foster a more immunosuppressive microenvironment. Of particular importance is the interaction between PD-1 blockade and macrophages, especially those involving Fcγ receptors (FcγRs).^347^ Studies by Dahan et al.^347^ and Lo Russo et al.^348^ highlight the negative impact of FcγR-mediated interactions with anti-PD-1 antibodies, which may not only dampen therapeutic efficacy but also promote tumor progression. Furthermore, recent findings by Zhang et al. on the PI3K/AKT/mTOR signaling pathway in macrophages, in the context of IL-4-induced metabolic shifts, add another layer of complexity.^349,350^ These shifts, including increased glycolysis and lactic acid production, alongside the upregulation of FcγRIIB expression, are thought to contribute to an immunosuppressive TME that disrupts CD8 + T cell function and accelerates tumor growth.^350^ Targeting FcγR interactions or modifying the Fc sequences of anti-PD-1 antibodies may present a promising strategy to mitigate HP and enhance the efficacy of ICIs. A deeper understanding of the intricate interplay between immune cells, receptors, and signaling pathways is crucial for optimizing immunotherapy approaches and addressing the challenges posed by HP.

### Prognosis and disease outcomes

#### Infinite Vulcan: Comprehensive rewiring of the Systemic Immune Repertoire

Persistent and prolonged antigen exposure not only establishes a highly inhibitory TME but also acts as a reservoir that facilitates the subsequent dissemination of cancer cells, thus leading to the anergy and exhaustion of the adaptive immune response, primarily through the induction of tumor-specific immune tolerance.^351^ These mechanisms substantially contribute to resistance to ICB therapy, initiating a cascade of deleterious effects.^174,352^ The cancer-immunity cycle—an iterative process encompassing tumor antigen release, dendritic cell-mediated presentation, T cell priming/activation, effector cell trafficking, and target cell elimination—has emerged as a foundational paradigm. Increasingly sophisticated models of the cancer-immunity cycle^190,353^ highlight the critical role of immune dysfunction, particularly due to the substantial accumulation of tumor-associated immune cells in lymphoid organs and the liver (Fig. 6). This growing understanding underscores the importance of these immune alterations in the broader context of cancer progression and therapeutic resistance.

Tumor-draining lymph nodes (tdLNs) are initially involved in immune surveillance prior to the onset of distant metastasis. In the complex TME, these tdLNs undergo profound transformation, adopting a tumor-specific immune-tolerant phenotype. This shift enables tdLNs to evade NK cell activity, impairs the generation of antitumor migratory Teff cells, and inhibits T cell-mediated cytotoxicity, diverging from their original sentinel function.^354,355^ Central to this process are antigen-specific regulatory T cells, which play a critical role in orchestrating immune tolerance within tdLNs.^356^ Moreover, the physicochemical environment of tdLNs is a key determinant in modulating both metabolic and epigenetic landscapes, thereby influencing subsequent immune responses. A well-established reciprocal relationship exists between acidity and glycolytic metabolism, with low pH conditions suppressing the activity of monocarboxylate and glucose transporters. This interplay is crucial for modulating T-cell activation.^357^ The accumulation of lactic acid and highly acidic lymphatic fluid in tumor-draining lymph nodes (tdLNs) leads to lymph node acidification, triggering a pH-dependent feedback mechanism that inhibits T-cell glycolysis and prevents full activation. This creates a state of T-cell anergy within tdLNs prior to the migration and establishment of cancer cells.^358^ Furthermore, as cancer cells colonize and proliferate, they reshape the local metabolic microenvironment, creating conditions of heightened hypoxia^359^ and acidity.^360^ These alterations further hinder immune priming within tdLNs, contributing to immune evasion by the tumor.^361^ In the complex metabolic environment surrounding tumors, a diverse range of tumor-specific CD8 + T cells exist, including subsets such as progenitors of exhausted T (Tpex) cells, terminally Tex cells, tdLN-derived tumor-specific memory (TTSM) cells,^352^ stem-like CD8 + T cells, and tissue-resident memory T (TRM) cells.^354^ Each of these subsets exhibits distinct epigenetic profiles, which significantly influence their pro- or antitumor activities and their responses to various immunotherapies. Together, these epigenetic and metabolic factors contribute to the development of a highly immunosuppressive TME in tdLNs.^352^ Additionally, lymphatic fluid in tdLNs contains higher concentrations of BAs^362^ and triacylglycerols than do those in phosphatidylcholines.^363^ BAs are particularly important in this context, as they serve as potential molecular triggers for the YAP-dependent metabolic shift toward FAO.^364^ This shift exacerbates the decline in local pH,^361^ and leads to increased FAO-derived H3K27ac levels,^365^ further hindering T-cell activation. These combined metabolic and epigenetic alterations promote a repressive microenvironment that impairs immune responses within tdLNs and contributes to immune evasion by the tumor^361^ (Fig. 6b). Notably, in glioblastoma, the cancer-immunity cycle is noncanonical and an optimal pathway, namely, the interstitial fluid (ISF)-cerebrospinal fluid (CSF)-meningeal lymphatic vessel (MLV)-lymph node (CLN) pathway, which facilitates communication from the CNS to the periphery. Current evidence suggests that soluble antigens and antigens loaded by APCs are primarily drained by MLVs in the dura mater from tumor sites to dCLNs.^366^ The lymphatic vasculature plays a crucial role in shaping the peripheral tolerance and functional state of the intratumoral CD8^+^ T-cell repertoire,^367^ and oncogenic metabolites may disrupt the antitumor immune response mediated by DCs.^368^ Collectively, tdLNs act as pivotal hubs in systemic immune circulation, where metabolic dysregulation and epigenetic reprogramming could reshape local immune cascades and ultimately rewire whole-body immune responses. These findings highlight the therapeutic potential of targeting metabolic‒epigenetic axes to restore nodal and systemic antitumor immunity.

Liver metastases are associated with systemic immunosuppression in preclinical models,^369^ and can serve as a clinical indicator of poor prognosis and a reduced response to anti-PD-1 immunotherapy.^370,371^ This is largely due to the unique immunological environment of the liver, which is characterized by an abundance of both conventional and nonconventional antigen-presenting cells. While this composition helps maintain immune tolerance, it also complicates the activation of robust immune responses against tumors. Liver metastases can lead to the systemic loss of antigen-specific T cells, further contributing to immune evasion.^372^ Several hepatic cell types have been implicated in the modulation of T cell fate and survival within the liver, including Kupffer cells,^373^ liver sinusoidal endothelial cells,^374^ hepatocytes,^375^ plasmacytoid dendritic cells,^376^ NKT cells,^286^ stellate cells,^377^ and tumor-infiltrating myeloid cells. Notably, research using a dual-tumor immunocompetent mouse model revealed that the systemic suppression of antitumor immunity is mediated by the coordinated activation of Treg cells and MDSCs.^369^ Unbiased scRNA-seq has also revealed that hepatic monocyte-derived CD11b + F4/80+ macrophages play a critical role in inducing antigen-specific CD8 + T-cell apoptosis via the Fas‒FasL pathway, thereby reducing peripheral T-cell numbers and further impairing antitumor immunity^370^ (Fig. 6c). Given that the liver is a metabolic organ, it is also exposed to metabolites from the gut microbiome, as well as products from the intestine and systemic circulation, which underscores the need for immune tolerance in this organ. The immune tolerance of the liver is closely associated with its metabolic milieu, highlighting the potential for modulating both metabolic and epigenetic pathways to restore systemic antitumor immunity. Epigenetic therapies, such as inhibitors of EZH2, have demonstrated efficacy in reactivating immune responses and enhancing the effectiveness of ICB.^369^ However, the interplay between metabolic and epigenetic pathways in this context remains inadequately understood. Further investigation is essential to delineate the complex, potentially synergistic or antagonistic, interactions between these pathways.

In summary, persistent crosstalk—encompassing molecular, metabolic, and mechanical interactions—between the evolving host and invading cancer cells likely plays a pivotal role in early tumor progression. This dynamic interplay may offer novel therapeutic targets for intercepting disease at disease onset and enhancing immune surveillance.^378^ Future research is expected to explore the roles of “LN-T cells” and “liver-T cells” as key factors in the development of more effective immunotherapy strategies for cancer patients.

## Therapeutic targeting of the metabolic-epigenetic-immune axis

Cancer cells have developed intricate cascade strategies to promote their survival, making the disruption of these mechanisms a critical component of effective cancer therapy. Direct targeting of the tumor immune microenvironment is pivotal; however, in tumors with a cold TME, the efficacy of immunotherapy remains limited. To overcome this challenge, leveraging metabolic^379^ and epigenetic interventions^22^ to reprogram the TME into a more immunologically active or “hot” state is a promising approach. However, owing to the inherent complexity of tumor biology, inhibiting specific metabolic or epigenetic pathways may inadvertently trigger compensatory resistance mechanisms.^380^ Therefore, a more refined approach is needed—one that targets critical nodes within the interconnected networks of metabolism, epigenetics, and immunity. Such a targeted strategy may more effectively disrupt tumor survival mechanisms and enhance treatment efficacy.

### Reprogramming metabolic pathways to potentiate cancer immunotherapy

The interplay between tumor metabolism and cancer immunity presents promising avenues for enhancing the efficacy of immunotherapy by targeting metabolic pathways simultaneously. In this context, we provide an overview of recent advancements in the combined use of immunotherapy and metabolism-related molecules, drawing from both preclinical studies and clinical trials, as shown in Table 1. Targeting key metabolic enzymes involved in cancer metabolism offers a novel strategy for increasing ICB efficacy. In addition, adoptive cell therapy (ACT), which harnesses tumor-reactive T cells for the targeted elimination of malignancies, can be augmented through metabolic modulation to increase therapeutic efficacy.^381^

**Table 1:** Combinations of immunotherapies with Metabolic Intervention

Metabolic Intervention	Immunotherapy	Conditions	NCT number	Stage	References (DOI)
LDHA knockdown (glycometabolism)	Anti-PD-1	Melanoma cell line	NA	Preclinical	10.3390/cancers11040450^384^
PFKFB3 inhibitor of PFK-158 (glycometabolism)	Anti-CTLA-4	Lung cancer cell line	NA	Preclinical	10.1158/1535-7163.MCT-13-0097^471^
Reduce hypoxia with metformin (glycometabolism)	Anti-PD-1	Melanoma and colon cancer cell line	NA	Preclinical	10.1158/2326-6066.CIR-16-0103^385^
Glycolytic inhibitor of 2-deoxyglucose (glycometabolism)	Adoptive cell therapy (CD8 + T cell)	Melanoma cell line	NA	Preclinical	10.1172/JCI69589^386^
Enhance OXPHOS with interleukin-10-Fc protein (glycometabolism)	Adoptive cell therapy (CD8 + T cell)	Melanoma, colorectal and ovarian cancer cell line	NA	Preclinical	10.1038/s41590-021-00940-2^387^
IDO inhibitors of PF-06840003 (amino acid metabolism)	Avelumab (anti-PD-L1)	Melanoma, glioblastoma, ovarian, cervical, breast cancer cell line	NA	Preclinical	10.1158/1535-7163.MCT-17-1104^395^
IDO inhibitors of EOS200271/PF06840003 (amino acid metabolism)	Avelumab (anti-PD-L1)	Malignant gliomas	NCT 02764151	Phase 1	10.1158/1535-7163.MCT-17-1104^395^
IDO inhibitors of BGB-5777 (amino acid metabolism)	Nivolumab (anti-PD-1)	Recurrent glioblastomas	NCT 02336165 NCT 02617589 NCT 02667587	Phase 1/2	10.1158/1078-0432.CCR-17-3573^394^
PRMT5 inhibitors of GSK591 (amino acid metabolism)	Anti-PD-L1	Lung cancer cell line	NA	Preclinical	10.3389/fimmu.2021.722188^392^
PMN-MDSC inhibitors of POG (amino acid metabolism)	Anti-PD-1	Breast cancer cell line	NA	Preclinical	10.1186/s40425-019-0676-z^472^
Administration of L-arginine (amino acid metabolism)	Adoptive cell therapy (CD8 + T cell)	Melanoma cell line	NA	Preclinical	10.1016/j.cell.2016.09.031^390^
Arginine deprivation using ADI-PEG20 (amino acid metabolism)	Pembrolizumab (anti-PD-1)	Nasopharyngeal carcinoma, melanoma, CRC, HCC, Cholangiocarcinoma	NCT 03254732	Phase 1	10.1080/2162402X.2021.1943253^473^
Arginase inhibitiors of CB-1158 (amino acid metabolism)	Pembrolizumab (anti-PD-1)	CRC and NSCLC	NCT 02903914	Phase 1	10.1093/annonc/mdz244.002^474^
Blockade glutamine with JHU083 (amino acid metabolism)	Anti-PD-1	Colon cancer, lymphoma and melanoma cell line	NA	Preclinical	10.1126/science.aav2588^389^
Glutamate deprivation using Trigriluzole (amino acid metabolism)	Nivolumab or pembrolizumab (anti-PD-1)	Melanoma, NSCLC, RCC, bladder/urothelial, ovarian cancer, adenoid cystic carcinoma, pleural mesothelial, head and neck cancer	NCT 03229278	Phase 1	10.1186/s40001-022-00732-w^475^
ACAT1 inhibitors of Avasimibe (cholesterol metabolism)	Anti-PD-1	Lymphoma, melanoma and lung carcinoma cell line	NA	Preclinical	10.1038/nature17412 Abstract^397^
Inhibiting adenosine via A2aR antagonist using AZD4635 (nucleic acid metabolism)	Anti-PD-L1	Fibrosarcoma, melanoma and colon carcinoma cell line	NA	Preclinical	10.1136/jitc-2019-000417^476^
A2AR antagonist CPI-444 using AZD4635 (nucleic acid metabolism)	Atezolizumab (anti-PD-L1)	RCC	NCT 02655822	Phase 1	10.1158/2159-8290.CD-19-0980^399^
A2AR antagonist CPI-444 using AZD4635 (nucleic acid metabolism)	Durvalumab (anti-PD-L1)	Metastatic castration resistant prostate cancer	NCT 02740985	Phase 1	10.1158/1538-7445.Am2019-ct026^477^

*RCC* renal cell carcinoma, *CRC* colon rectal carcinoma, *HC*C hepatocellular carcinoma, *NSCLC* non-small cell lung cancer, *NA* not applicable

As mentioned above, lactate-induced activation of PD-L1 in tumor cells is a key mechanism underlying immune suppression,^382^ and the expression of LDHA also increases NAD+ levels to regulate PD-L1 expression.^383^ Interestingly, in murine melanoma models, the inhibition of LDHA enhances the effectiveness of anti-PD-1 therapy.^384^ Additionally, metformin has been shown to sensitize patient-derived xenograft (PDX) models to ICB by alleviating the hypoxic TME.^385^ Moreover, inhibiting glycolysis while activating CD8 + T cells enhances the formation of long-lived memory T cells, which boosts their tumoricidal function.^386^ In addition to glycolysis, Guo et al. demonstrated that IL-10/Fc mediates T-cell metabolic reprogramming by promoting oxidative phosphorylation, thus rejuvenating exhausted T cells and improving responses to cancer immunotherapy.^387^ Additionally, studies have shown that inhibiting glycogen synthase kinase-3 (GSK-3) activity with small molecules can reduce PD-1 levels, thereby increasing the cytotoxic potential of CD8 + T cells.^388^

Amino acid metabolism has also emerged as a critical target for enhancing immunotherapy. Notably, the glutaminase inhibitor JHU083 effectively halted these metabolic processes in murine cancer cells while simultaneously promoting OXPHOS in T cells. This dual action significantly increases the efficacy of PD-1 antibody therapy in combination with JHU083.^389^ In a separate approach, the administration of L-arginine to B16-OVA tumor-bearing mice enhances the activation of OT-I T cells, resulting in improved tumor control.^390^ Protein arginine methyltransferase 5 (PRMT5), a key enzyme involved in arginine methylation, regulates critical processes such as RNA splicing and the DNA damage response.^391^ Inhibition of PRMT5, combined with anti-PD-L1 therapy, has been shown to increase the number of tumor-infiltrating T cells and enhance their functional capacity, thus improving the outcome of lung cancer.^392^ Additionally, indoleamine 2,3-dioxygenase (IDO), an enzyme responsible for tryptophan degradation, leads to the production of N-formyl-kynurenine, which can impair immune responses.^393^ IDO inhibitors, such as navoximod, epacadostat, linrodostat, and indoximod, are being explored as immunomodulatory agents, either alone or in combination with other cancer therapies, to enhance antitumor immunity.^394,395^

In the context of lipid metabolism, the metabolic reprogramming of CD8 + T cells through increased fatty acid catabolism has been shown to improve the antitumor efficacy of ACT for enhanced tumor elimination.^396^ Research by Wei Yang and colleagues further demonstrated that inhibition of acyl-CoA:cholesterol acyltransferase 1 (ACAT1) activity leads to increased cholesterol levels in CD8 + T-cell membranes, thereby increasing T-cell signaling and promoting the formation of more effective immune synapses.^397^ ACAT inhibitors, such as avasimibe, are cholesterol-modulating agents that have been well tolerated in clinical trials as cholesterol-lowering drugs.^398^ Preliminary studies suggest that combining avasimibe with PD-1 antibodies may increase the efficacy of tumor immunotherapy.^397^ Additionally, the combination of A2A receptor (A2AR) antagonists with anti-PD-L1 antibodies has been shown to increase CD8 + T-cell recruitment, demonstrating promising antitumor activity in refractory renal cell carcinoma.^399^ Targeting key metabolic nodes involved in carbohydrate, lipid and amino acid metabolism is promising for reprogramming the immunosuppressive TME and sensitizing tumors to ICI or ACT therapies. However, systematic studies to uncover combinatory strategies to coopt metabolic‒immune crosstalk for enhanced therapeutic efficacy have yet to be performed.

### Targeting the epigenetic regulation of antitumor immunity

The potential of epigenetic therapy to facilitate immune recognition of tumor cells, not least through the augmentation of antigen expression, processing and presentation, has been demonstrated, as mentioned above. The following sections summarize the evidence accumulated to date that epigenetic therapy can overcome barriers to clinical responses to immunotherapy, including ICI and ACT, as listed in Table 2.

**Table 2:** Combinations of immunotherapy with Epigenetic Intervention

Epigenetic targets	Immunotherapy	Conditions	NCT number	Stage	References
Decitabine and azacitidine (DNMTi)	Anti-CTLA-4	Ovarian Cancer and melanoma cell line	NA	Preclinical	10.1158/2326-6066.CIR-15-0073^478^ 10.1016/j.cell.2015.07.011^479^
Azacytidine (DNMTi)	Ipilimumab (anti-CTLA4)	Myelodysplastic Syndromes	NCT 02530463	Phase 2	10.1038/s41375-024-02457-7^480^
Guadecitabine (DNMTi)	Anti-PD-1	Breast cancer cell line	NA	Preclinical	10.1038/s41467-017-02630-w^405^
Decitabine (DNMTi)	Anti-PD-1	CRC cell line	NA	Preclinical	10.1038/s41423-018-0026-y^406^
Azacytidine (DNMTi)	Nivolumab (anti-PD-1)	AML	NCT 02397720; NCT 03825367	Phase 1/2	10.1038/s41467-021-26282-z^481^ 10.3390/cancers16030496^482^
Azacytidine (DNMTi)	Nivolumab (anti-PD-1)	NSCLC	NCT 01928576	Phase 2	10.1016/j.cell.2015.08.005^483^
Azacytidine (DNMTi)	Nivolumab (anti-PD-1)	Osteosarcoma	NCT 03628209	Phase 1/2	10.1016/j.canlet.2022.215887^484^
Azacytidine (DNMTi)	Pembrolizumab (anti-PD-1)	Ovarian cancer	NCT 02900560	Phase 2	10.1016/j.semcancer.2020.10.016^485^
Decitabine (DNMTi)	Camrelizumab (anti-PD-1)	Hodgkin lymphoma	NCT 03250962	Phase 2	10.1136/jitc-2021-002347^486^
Decitabine (DNMTi)	Nibolumab (anti-PD-1)	NSCLC	NCT 02664181	Phase 2	10.1200/JCO.2018.36.15_suppl.e24134^487^
Decitabine (DNMTi)	Anti-PD-L1	Prostate adenocarcinoma cell line	NA	Preclinical	10.1016/j.cell.2017.06.007^407^
Decitabine (DNMTi)	Avelumab (anti-PD-L1)	AML	NCT 03395873	Phase 1	10.1002/ajh.26043^488^
Panobinostat (HDACi)	Anti-PD-1	Melanoma cell line	NA	Preclinical	10.1158/2326-6066.CIR-15-0077-T^409^
Entinostat (HDACi)	Anti-PD-1	Lung carcinomaand RCC cell line	NA	Preclinical	10.1158/1078-0432.CCR-17-0741^408^
Entinostat (HDACi)	Pembrolizumab (anti-PD-1)	respiratory tract, digestive system, and endocrine gland neoplasms, RCC	NCT 02909452	Phase 1	10.1016/j.critrevonc.2018.07.001^489^
Entinostat (HDACi)	Pembrolizumab (anti-PD-1)	SCLC, melanoma, CRC	NCT 02437136	Phase 1/2	10.3390/cancers14010066^490^
Entinostat (HDACi)	Pembrolizumab (anti-PD-1)	Metastatic uveal melanoma	NCT 02697630	Phase 2	10.3779/j.issn.1009-3419.2021.102.11^491^
ACY 241 (HDAC-6 i)	Nivolumab (anti-PD-1)	NSCLC	NCT 02635061	Phase 1	10.3389/fonc.2021.696512^492^
Entinostat (HDACi)	Avelumab (anti-PD-L1)	Ovarian, peritoneal, fallopian tube cancer	NCT 02915523	Phase 1/2	10.1200/JCO.2019.37.15_suppl.5511^493^
Entinostat (HDACi)	Atezolizumab (anti-PD-L1)	Breast cancer	NCT 02708680	Phase 1	10.1200/JCO.2020.38.15_suppl.1014^494^
Mocetinostat (HDACi)	Durvalumab (anti-PD-L1)	Advanced cancer	NCT 02805660	Phase 1/2	10.1016/j.cllc.2023.01.013^495^
Panobinostat (HDACi)	Ipilimumab (anti-CTLA4)	Stage III or IV melanoma	NCT 02032810	Phase 1	10.1016/j.cllc.2023.01.013^495^
Belinostat (HDACi)	Anti-CTLA4	HCC cell line	NA	Preclinical	10.1007/s00262-018-2283-0^411^
GSK503 (EZH2i)	Anti-CTLA4	Melonoma cell line	NA	Preclinical	10.1016/j.celrep.2017.07.007^412^
CPI-1205 (EZH2i)	Anti-CTLA4	Lung adenocarcinoma	NCT 03525795	Phase 1	10.1053/j.seminoncol.2022.06.005^496^
HCI-2509 (LSDi)	Anti-PD-1	Breast cancer cell line	NA	Preclinical	10.1038/s41388-018-0451-5^413^
JQ-1 (BETi)	Anti-PD-1	Mouse models of NSCLC	NA	Preclinical	10.1158/2326-6066.CIR-18-0077^414^

*AML* acute myeloid leukemia, *RCC* renal cell carcinoma, *CRC* colon rectal carcinoma, *HCC* hepatocarcinoma, *SCLC* small cell lung cancer, *NA* not applicable

Direct evidence supporting the synergy between DNMT inhibition and ICB has been established across multiple preclinical models. In animal models of ovarian cancer and melanoma, the combination of demethylating agents, such as decitabine and azacitidine, with anti-CTLA-4 antibody therapy has been shown to enhance antitumor responses.^400–404^ In a mouse breast cancer model, treatment with guadecitabine significantly increased MHC-I expression and promoted T-cell chemotaxis to potentiate responses to anti-PD-1 therapy.^405^ Furthermore, Yu et al. demonstrated key immunological effects of decitabine in a colon cancer mouse model, including activation of antigen presentation mechanisms, accumulation of PD-1 + CD8 + T cells within the tumor, and heightened sensitivity to anti-PD-1 therapy.^406^ In a mouse model of ICI-resistant prostate cancer, decitabine treatment was found to restore CD8 + T cell sensitivity to anti-PD-L1 antibodies by preventing DNMT3A-driven DNA methylation in exhausted T cells, thereby enhancing antitumor immunity.^407^ In addition, in preclinical models of solid tumors, HDAC inhibitors (HDACis), including entinostat, panobinostat, romidepsin and belinostat, have been demonstrated to enhance the efficacy of ICIs. Specifically, entinostat has been shown to deplete MDSCs, thereby improving responses to anti-PD-1 therapy.^408^ Similarly, panobinostat potentiates anti-PD-1 treatment, resulting in slower tumor progression and prolonged survival.^409^ Romidepsin, through the upregulation of T cell chemoattractants and increased tumor infiltration, sensitizes lung adenocarcinoma tumors to anti-PD-1 therapy.^410^ Moreover, in a subcutaneous mouse model of hepatocellular carcinoma, belinostat increased the effectiveness of CTLA-4 inhibition, which was accompanied by an increase in M1-polarized TAMs, increased IFN-γ production by CD8 + T cells, and a reduction in splenic Treg cells.^411^ Additionally, the inhibition of EZH2 has been shown to reverse adaptive resistance mechanisms, thereby enhancing the efficacy of anti-CTLA-4 therapy in melanoma models.^412^ Similarly, LSD1 inhibitors elicit a viral mimicry-like response that sensitizes immunologically cold tumors, such as TNBC, to anti-PD-1 therapy.^161,413^ Furthermore, JQ1, a bromodomain inhibitor, has demonstrated synergy with anti-PD-1 antibodies in non-small cell lung cancer models harboring *Kras* mutations and *Tp53* deletions.^414^ These preclinical findings underscore the potential of combining ICIs with agents targeting DNA methylation, BET proteins, LSD1, or EZH2, a strategy currently being explored in ongoing clinical trials, as shown in Table 1.

Epigenetic therapies, including chimeric antigen receptor T (CAR-T) cell therapy, are also gaining recognition for their potential to increase the efficacy of ACT.^415^ HDAC inhibitors, for example, have been shown to improve ACT by promoting the trafficking of transferred T cells to tumor sites.^416^ Recent studies have identified the histone demethylase TET2 as a promising immunomodulatory target that preserves CAR-T cells in a central memory-T phenotype,^417^ and is more effective than the use of conventional Teff cells.^418^ Moreover, several patent studies have suggested that DNA demethylase-LSD1 inhibitors can be applied during CAR-T cell manufacturing to increase antitumor efficacy.^419^ In contrast, the H3K9 methyltransferase SUV39H1, which plays a crucial role in the transition from naive to effector T cells, represents another promising target for epigenetic modulation within ACT settings.^420^ Additionally, bromodomain and extraterminal (BET) inhibitors, such as JQ1, have been shown to prevent the transition of T cells to an effector memory phenotype, a process that, in turn, enhances the functional capacity of anti-CD19 CAR-T cells in vivo.^421^ Collectively, these findings highlight the transformative potential of epigenetic therapies in optimizing CAR-T-cell manufacturing and suggest that continued refinement of these strategies may enable the selective enhancement of T-cell properties, thereby improving therapeutic outcomes.

### Upstream regulatory control of this axis

**Fig. 7:**
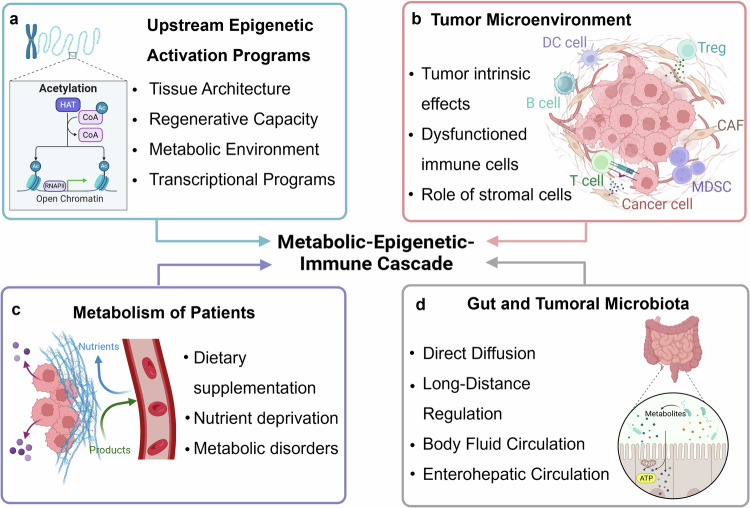
Strategies to modulate the metabolic‒epigenetic-immune axis. Intervening in the metabolic‒epigenetic-immune axis can be approached through four main avenues: **a** targeting the upstream epigenetic mechanisms that govern metabolic processes; **b** directly modulating the tumor microenvironment; **c** implementing dietary interventions to influence metabolic pathways; and **d** regulating the composition of the microbiome. The figure was generated with BioRender (https://biorender.com). DC dendritic cell, CAF cancer-associated fibroblast; Treg, regulatory T cell; MDSC, myeloid-derived suppressor cell

In addition to targeting key nodes within the cascade mechanism, macroregulation of the upstream components of the cascade axis represents a crucial strategy (Fig. 7). Various signaling pathways play pivotal roles in regulating metabolism, thereby influencing downstream cascades. These include the NF-κB,^60^ TNF-α,^422^ PI3K/AKT/mTOR,^423^ IL-6/JAK/STAT3,^424^ MAPK,^425^ and PI3K,^426^ pathways, each of which contributes to the modulation of cellular processes that are integral to metabolic homeostasis. Notably, epigenetic reprogramming plays a pivotal role in initiating metabolic remodeling (Fig. 7a). Recent studies have demonstrated that three-dimensional genome rearrangements can drive alterations in genes involved in lipid and glucose metabolism^427^; silencing HDAC2 in hepatocellular carcinoma (HCC) cells markedly inhibits key regulators of glycolysis, such as ChREBPα and GLUT4, as well as lipogenesis, including SREBP1C and FAS^428^; JMJD1A has been shown to promote urothelial bladder cancer progression by enhancing glycolysis through the coactivation of HIF-1α,^429^; and FLI1 orchestrates the expression of CBP and STAT1, facilitating chromatin accessibility of IDO1, resulting in increased synthesis of Kyn in tumor cells.^430^ The onset of such reprogramming is intricately linked to local tissue architecture, metabolic conditions, and transcriptional networks. For example, liver metastases of pancreatic cancer^427^ often exhibit transcriptional signatures resembling those of the liver,^431^ with a predominant shift toward glycolysis. In contrast, lung metastases tend to favor oxidative phosphorylation.^432^ Similarly, the peritoneum, which is rich in adipocytes, presents a unique challenge for metastatic lesions, which must adapt to the surrounding fat cells. As a result, insulin-like growth factor 1 (IGF-1), a critical regulator of lipid metabolism, is notably upregulated in peritoneal metastases, reflecting the epigenetic adaptations that enable these lesions to thrive within their specific microenvironment.^433^ Given these insights into tissue-specific mechanisms, a more targeted approach to epigenetic pathways could offer a promising strategy for disrupting upstream signaling cascades and combating metastasis.

In addition to endogenous metabolic changes (Fig. 7b), dietary interventions have proven effective in mitigating the side effects associated with metabolic enzyme inhibition (Fig. 7c). Caloric restriction and fasting-mimicking diets have emerged as promising strategies for reprogramming metabolism to enhance antitumor immune responses. These interventions are believed to modulate key metabolic pathways, which, in turn, influence immune cell function and tumor progression. Notably, caloric restriction has been shown to reduce the expression of heme oxygenase-1 (HO-1), a factor that induces apoptosis in tumor cells and inhibits the activity of Treg cells.^434^ Similarly, carbohydrate restriction, through the elevation of circulating ketone bodies, suppresses the expression of PD-L1 on monocytes, thereby mitigating their immunosuppressive functions.^138^ Additionally, protein restriction decreases the serum levels of insulin-like growth factor 1 (IGF-1), leading to the inhibition of the IGF-1/IGF receptor 1 (IGFR1)/PI3K/AKT/mTORC1 signaling axis in TAMs and Treg cells.^435^ Collectively, these metabolic alterations foster an immune microenvironment that inhibits tumor cell proliferation and enhances anti-neoplastic immunity.^436^ Thus, caloric restriction, when employed as an adjunct to immunotherapy and chemotherapy, has potential for increasing therapeutic efficacy.

On the other hand, dietary creatine supplementation has been shown to inhibit tumor progression.^437^ As a “molecular battery,” creatine stores energy that is utilized by T cells to counteract the metabolic stress imposed by tumor cells. In support of this, tumor-infiltrating lymphocytes (TILs) upregulate the expression of creatine transporters, such as SLC6A8, facilitating the uptake of creatine and increasing the energy availability required for immune cell function.^438^ Numerous dietary therapies have been safely employed for various diseases.^439^ However, the influence of barriers and concentration gradients of the TME on the efficacy of these interventions remains poorly understood.

Metabolic dysregulation is also a critical factor in metabolic regulation and corresponding cascade signaling (Fig. 7c). The lipotoxic milieu associated with obesity drives the upregulation of PPARαδ genes, which promote lipid accumulation in NK cells—essential mediators of the antitumor immune response.^440^ In patients with obesity and breast cancer, adipocytes secrete elevated levels of fatty acids and adipokines, such as leptin, thereby facilitating tumor progression governed by STAT3.^441^ STAT3 reprograms cellular metabolism by enhancing FAO and suppressing glycolysis in Teff cells, which in turn impairs the antitumor immune response.^442^ This glycolytic inhibition, driven by STAT3, is associated with reduced secretion of IFN-γ and other T helper 1 (Th1) cytokines. Thus, the leptin-STAT3-FAO axis plays a pivotal role in linking obesity to compromised antitumor immunity.^443^ These studies illustrate that the systemic metabolic status could have a profound effect on local TME metabolic bias and further regulate immunometabolic dynamics.

Concurrently, growing research on the microbiome and its metabolites has underscored their critical role in tumor progression,^193^ and immunotherapy responses,^444^ (Fig. 7d). Notably, Jia et al. recently demonstrated that the gut microbiota, particularly Lactobacillus jensenii (L.j.) and Clostridium scindens (C.s.), can synthesize IPA, which promotes the infiltration of CD8 + T cells into the TME. This process is further facilitated by histone acetylation, which enhances the modulation of Tpex and Teff cell populations, thereby increasing tumor sensitivity to ICB therapy.^445^ This discovery reveals a complex microbiota-metabolism-epigenetics-immunity axis in cancer.^446^ Additionally, the identification of microbial metabolites such as butyrate^447^ and indole-3-lactic acid^239^ as direct modulators of the epigenetic landscape in immune cells, particularly CD8 + T cells, represents a significant advancement in the field of immunology. The challenge now lies in harnessing strategies such as fecal microbiota transplantation, antibiotic treatments, and other approaches^448^ to disrupt pathogenic cascades while maintaining the delicate balance of the microbiome—a subject that warrants further investigation.

### Challenges and limitations

The combination of metabolic interference or epigenetic modulators with immunotherapy faces several enduring challenges. These include the intrinsic complexity and heterogeneity of cancer cells, the evolving repertoire of resistance mechanisms, and the pharmacokinetic properties of therapeutic agents, such as their half-life and selectivity. Addressing these issues is crucial for enhancing the efficacy and durability of combination therapies.

First, distinct types of tumors exhibit varying metabolic pathways and epigenetic profiles. This variability not only limits the universal applicability of most therapeutic strategies but also fosters the development of complex compensatory mechanisms that contribute to therapeutic resistance. Furthermore, a central issue in this combined approach is limited selectivity, which can be viewed from two key perspectives. First, epigenetic regulators and metabolic enzymes are widely distributed and exhibit both intercellular and intracellular heterogeneity. This broad distribution means that therapeutic interventions targeting these pathways within different TMEs may yield divergent outcomes for both tumor and immune cells. For example, although the glucose analog 2-deoxyglucose is effective in cancer cells, it has been shown to impair T cell metabolism, thereby reducing their antitumor function.^449^ Even worse, epigenetic interventions may have opposing effects within a single cell type. For example, EZH2 promotes T cell activation following TCR stimulation, and the EZH2+ subset of CD8 + T cells exhibits increased cytotoxicity.^450^ These findings suggest that while an EZH2 inhibitor could increase CD8 + T cell infiltration, it may also limit CD8 + T cell functional activity. Therefore, identifying the most critical and proper metabolic or epigenetic event to target in a specific cancer type will be vital for optimizing therapeutic strategies. Recent advances, such as the use of scRNA-seq to identify cell subpopulations, offer a promising avenue for assessing the impact of various therapies at a relatively high resolution.^451^ The development of novel platforms capable of delivering metabolic or epigenetic reprogramming agents in a cell type-specific manner will be essential for achieving selective targeting. In addition, given the isoform-selective inhibition of epigenetic regulators and metabolic enzymes, research efforts should prioritize the investigation of the specific and detailed effects of different inhibitors. A recent study revealed that HDAC3 plays a critical role in negatively regulating a cytotoxic effector-associated transcriptional program in CD8 + T cells. However, the small-molecule HDAC3-selective inhibitor RGFP966 has been shown to significantly enhance the cytotoxic function of CD8 + T cells.^452^ Thus, the optimal dosing and treatment schedules for combining ICIs with epigenetic and metabolic therapies remain uncertain, posing a key pharmacokinetic and pharmacodynamic challenge. Interestingly, the advent of the AlphaFold platform presents a promising opportunity to elucidate the mechanisms underlying the interactions between small-molecule inhibitors and their target proteins, thereby facilitating the understanding, identification, and modification of molecules for enhanced specificity and targeted therapeutic applications.

Finally, a significant barrier to the clinical translation of immunotherapy lies in the discrepancies between mouse models and human conditions,^453^ particularly with respect to cancer metabolism and epigenetic profiles. This challenge complicates the translation of effective combination therapies observed in preclinical studies to successful clinical outcomes. To address this issue, diverse preclinical models, including genetically engineered mouse models, PDX models and patient-derived organoid (PDO) models, which more accurately reflect human malignancies and enable better assessment of immunotherapy responses, are essential. In addition, while immunotherapies have shown remarkable success in treating hematological malignancies, their efficacy in solid tumors remains limited. Biological differences in gene regulation between solid and hematological tumors add another layer of complexity to the clinical translation of these therapies.

Future studies must focus on elucidating the mechanistic links between immunotherapy and the metabolic or epigenetic landscapes of both cancer and immune cells. Key areas of investigation should include the identification of therapeutic windows for optimized interventions and the potential involvement of metabolic and epigenetic reprogramming in tumor resistance to immunotherapies, including ICB and ACT.

## Conclusion and perspective

The intricate interplay between metabolic rewiring, epigenetic plasticity, and immune evasion defines the adaptive resilience of cancer, as encapsulated in our proposed “metabolism‒epigenetic‒immune axis”. This framework not only elucidates the spatiotemporal dynamics of tumor progression but also challenges conventional therapeutic paradigms by emphasizing the need for multidimensional targeting. Below, we contextualize this regulatory axis within broader biological and clinical landscapes, addressing key unresolved questions and proposing actionable strategies to advance precision oncology.

### Spatiotemporal metabolic-epigenetic regulation of immune landscapes

This framework posits that organ-specific metastatic tropism is shaped by metabolic‒epigenetic crosstalk tailored to the local microenvironment of target organs. Such divergence underscores the need for spatially resolved diagnostics. Advances in metabolic imaging (e.g., DESI-MSI for lactate mapping),^454^ and epigenomic profiling (e.g., scNano-HiC for 3D chromatin architecture),^455^ now enable visualization of these interactions in situ. However, subcellular resolution remains elusive—quantum dot-labeled metabolic probes (e.g., 2-NBDG for glucose uptake,^456^; FiLa for lactate production,^457^) coupled with ATAC-see technology,^458^ could map metabolite‒chromatin colocalization to some extent. This would clarify how metabolic gradients directly sculpt promoter‒enhancer interactions in cancer or immune cells, informing spatially targeted therapies. Furthermore, integrating these tools with single-cell multi-omics could be used to dissect clonal evolutionary trajectories and identify metastasis-specific vulnerabilities.^459^

### Utilizing the metabolic-epigenetic-immune axis to analyze heterogeneity

Tumor heterogeneity manifests at two interdependent levels: interpatient heterogeneity, which reflects divergent genetic, environmental, and immunological backgrounds across individuals, and intratumor heterogeneity, which arises from clonal evolution and microenvironmental adaptation within a single tumor ecosystem.^460^ The conventional molecular taxonomy of cancers, largely anchored in genomic and transcriptomic features,^461^ often fails to capture the dynamic functional states shaped by metabolic-epigenetic-immune crosstalk. This framework posits that incorporating three dimensions into subtyping paradigms could refine prognostic accuracy and risk stratification. Crucially, this approach also helps address intratumoral heterogeneity. Single-cell multi-omics has revealed coexisting subclones with divergent metabolic dependencies (Warburg vs. reverse Warburg),^462^ and epigenetic immune editing capacities.^463^ Deconvolving these subsets—e.g., identifying mIDH clones that convert α-KG to D-2HG to increase H3K27me3,^464^ versus OXPHOS-dependent clones that exhibit increased succinyl-CoA and PD-L1 levels,^465^—this approach not only illuminates why histologically identical tumors respond disparately to therapy but also provides actionable biomarkers to guide the design of “combinatorial niche-targeted” therapies.

### Sequential and coordinated approaches in multitarget therapies

The metabolic‒epigenetic‒immune axis operates as a dynamic, phase-shifting network, raising critical questions about the optimal timing and sequence of therapeutic interventions. Notably, such sequential approaches should be tailored to organ-specific metabolic characteristics. Emerging evidence suggests that glycolytic suppression in primary tumors^100^ and liver metastases^132^ may reduce histone lactylation, thereby attenuating Treg infiltration and restoring CD8 + T cell cytotoxicity. Conversely, OXPHOS-dominated pulmonary metastases may resist glycolysis-targeted agents but respond to α-ketoglutarate (α-KG) supplementation to sustain TET2 activity.^466^ To operationalize this, real-time monitoring via hyperpolarized ^13^C-MRI (tracking lactate/α-KG flux)^467^ combined with liquid biopsy-based cfDNA hydroxymethylation profiling,^468^ could help to dynamically guide intervention timing. A pressing challenge lies in defining context-dependent therapeutic windows — whether metabolic normalization should precede or follow epigenetic reprogramming in specific metastatic niches — a question best addressed through adaptive clinical trial designs integrating multimodal biomarkers.

### Decoding multidimensional networks via AI-driven integration

The deluge of single-cell multi-omics data demands novel computational frameworks to identify nodal points within the metabolism‒epigenetic‒immune axis. Artificial intelligence (AI)-driven predictive platforms trained on pan-cancer multilayer datasets (histone modification states, oncometabolite flux, and TCR clonality) could predict evolutionary bottlenecks and optimize combinatorial regimens to dismantle the tumor immune shield. Critically, 3D microfluidic organoid platforms, which incorporate stromal and immune components, can model real-time metabolic-epigenetic-immune feedback and validate computational predictions. This framework provides preliminary screening and mechanistic validation for drug candidates entering clinical trials, accelerating translational progress.

### Evolutionary conservation of metabolic–epigenetic homeostasis

This review synthesizes extensive evidence establishing metabolic‒epigenetic interplay as a cornerstone of tumorigenesis across stages—from malignant transformation and immune evasion to metastatic adaptation and therapy resistance in humans. Strikingly, such coordination extends beyond humans, revealing evolutionarily conserved mechanisms that elevate the metabolic‒epigenetic equilibrium to a universal axis of cancer restraint. Naked mole rats, which are resistant to carcinogenesis, exhibit elevated hyaluronan synthase activity, which stabilizes genomic integrity via metabolic‒epigenetic coupling,^469^ while elephants leverage TP53 retrogenes to increase resistance to high-temperature metabolism coupled with cellular oxidative stress, increasing the probability of mutations.^470^ By framing metabolic‒epigenetic coordination through an evolutionary lens, we transcend species-specific adaptations to uncover universal vulnerabilities. This approach not only illuminates why certain pathways are recurrently hijacked in human cancers but also positions cross-species comparative oncology as a discovery engine for next-generation therapies.

In summary, the metabolism‒epigenetic‒immune axis redefines cancer as a dysregulated ecosystem where spatial, temporal, and evolutionary forces converge. Targeting this axis demands a paradigm shift from static, organ-agnostic therapies to dynamically adaptive strategies informed by real-time multi-omics. By integrating AI-driven biomarker discovery, synthetic microbial engineering, and quantum-enhanced imaging, we can decipher the hierarchical logic of tumor resilience, ultimately delivering precision therapies that outmaneuver the evolution of cancer. It requires concerted collaboration among clinicians, computational biologists, and translational scientists.
